# Understanding effective post‐test linkage strategies for HIV prevention and care: a scoping review

**DOI:** 10.1002/jia2.26229

**Published:** 2024-04-11

**Authors:** Beatrice Wamuti, Muhammad S. Jamil, Nandi Siegfried, Nathan Ford, Rachel Baggaley, Cheryl Case Johnson, Peter Cherutich

**Affiliations:** ^1^ Department of Global Health and Population Harvard University Cambridge Massachusetts USA; ^2^ Global HIV, Hepatitis and STIs Programs, World Health Organization Geneva Switzerland; ^3^ Regional Office to the Eastern Mediterranean, World Health Organization Cairo Egypt; ^4^ Independent Clinical Epidemiologist Cape Town South Africa; ^5^ Department of Preventive and Promotive Health Ministry of Health Nairobi Kenya

**Keywords:** ARV, HIV care continuum, HIV prevention trials, HIV prevention, linkage to care, treatment

## Abstract

**Introduction:**

Following HIV testing services (HTS), the World Health Organization recommends prompt linkage to prevention and treatment. Scale‐up of effective linkage strategies is essential to achieving the global 95‐95‐95 goals for maintaining low HIV incidence by 2030 and reducing HIV‐related morbidity and mortality. Whereas linkage to care including same‐day antiretroviral therapy (ART) initiation for all people with HIV is now routinely implemented in testing programmes, linkage to HIV prevention interventions including behavioural or biomedical strategies, for HIV‐negative individuals remains sub‐optimal. This review aims to evaluate effective post‐HTS linkage strategies for HIV overall, and highlight gaps specifically in linkage to prevention.

**Methods:**

Using the five‐step Arksey and O'Malley framework, we conducted a scoping review searching existing published and grey literature. We searched PubMed, Cochrane Library, CINAHL, Web of Science and EMBASE databases for English‐language studies published between 1 January 2010 and 30 November 2023. Linkage interventions included as streamlined interventions—involving same‐day HIV testing, ART initiation and point‐of‐care CD4 cell count/viral load, case management—involving linkage coordinators developing personalized HIV care and risk reduction plans, incentives—financial and non‐financial, partner services—including contact tracing, virtual—like social media, quality improvement—like use of score cards, and peer‐based interventions. Outcomes of interest were linkage to any form of HIV prevention and/or care including ART initiation.

**Results:**

Of 2358 articles screened, 66 research studies met the inclusion criteria. Only nine linkage to prevention studies were identified (*n* = 9/66, 14%)—involving pre‐exposure prophylaxis, voluntary medical male circumcision, sexually transmitted infection and cervical cancer screening. Linkage to care studies (*n* = 57/66, 86%) focused on streamlined interventions in the general population and on case management among key populations.

**Discussion:**

Despite a wide range of HIV prevention interventions available, there was a dearth of literature on HIV prevention programmes and on the use of messaging on treatment as prevention strategy. Linkage to care studies were comparatively numerous except those evaluating virtual interventions, incentives and quality improvement.

**Conclusions:**

The findings give insights into linkage strategies but more understanding of how to provide these effectively for maximum prevention impact is needed.

## 1 INTRODUCTION

Globally, linkage to HIV services, defined as a process to support people testing for HIV to engage with prevention, care, treatment and other relevant non‐HIV‐related services, is an important component of HIV testing [1]. However, achieving effective linkage remains a challenge to achieving the 95–95–95 targets [2]. People living with HIV (PLHIV) who are not on antiretroviral therapy (ART) continue to drive new HIV acquisitions. To address this gap, in 2019, the World Health Organization (WHO) updated its Consolidated Guidelines and recommended that HIV testing services (HTS) employ the following effective linkage strategies: (1) support for HIV disclosure; (2) case management; (3) partner and social network testing services; (4) demand creation; (5) peer support and navigation; and (6) quality improvement (QI) approaches [3, 4, 5, 6].

Effective linkage is important as PLHIV on ART who are engaged in care and virally suppressed prevent onward HIV transmission and have improved quality of life [7, 8, 9]. Modelling suggests that prompt linkage to ART could reduce HIV incidence by 54% and mortality rate by 64% in the United States [10], and is further estimated that with 92% linkage to ART, HIV incidence would decline from 2.5% to 0.03% within 30 years in South Africa [11]. Therefore, WHO provides HIV guidance that outlines appropriate services to be offered according to local epidemiology, individual HIV risk and client needs [12].

In addition, HIV‐negative individuals with a high ongoing risk for HIV may benefit from a range of prevention interventions, such as voluntary medical circumcision (VMMC), partner services and pre‐exposure prophylaxis (PrEP) [13, 14, 15, 16]. Due to the diversity of prevention interventions, linkage strategies also need to be tailored as not all people testing negative require onward services [17]. Evidence to support effective HIV prevention linkage strategies remains limited and often not prioritized. Challenges such as lack of evidence and competing priorities impede the achievement of the UNAIDS target for 95% of people with HIV risk to receive combination prevention [2]. Consequently, implementation of linkage to prevention services is suboptimal, leaving many with high HIV risk unreached. Furthermore, integration of linkage strategies following HTS may be useful for other conditions, such as sexually transmitted infections (STIs), viral hepatitis and non‐communicable diseases (e.g. hypertension and cancers).

We, therefore, conducted a scoping review to assess strategies and best practices for linkage to HIV prevention (main objective) and care (secondary objective). This review provides a summary of the evidence, as well as critical implementation and research gaps.

## 2 METHODS

### 2.1 Study design

We conducted this scoping review following the Preferred Reporting Items for Systematic Reviews and Meta Analyses—Extension for Scoping Reviews (PRISMA‐ScR) checklist (Appendix S1) [18]. We utilized the Arksey and O'Malley framework [19], where these five steps were followed: (1) identifying a clear research objective and search strategies; (2) identifying relevant research articles; (3) selection of research articles; (4) extraction and charting of data; and (5) summarizing, discussing, analysing and reporting the results.

### 2.2 Literature search strategy

We searched the following online databases for literature published between 1 January 2010 and 30 November 2023: PubMed, Cochrane Library, CINAHL, Web of Science and EMBASE. We developed a search string using the appropriate MeSH terms supported by free‐text formats in the PubMed search builder. The final search string consisted of the core concepts (HIV Testing) AND (Linkage/Prevention/Care). The search string was then adapted to the other electronic databases (Appendix S1).

We also conducted a search of the grey literature directed at 19 major international funders and advocacy organizations involved with HIV programming identified as potential repositories (e.g. The Global Fund, Bill & Melinda Gates Foundation and Treatment Action Campaign). We searched trial registries for ongoing or completed studies and contacted investigators to obtain results that were close to publication. Linkage data were also solicited from national programmes, key experts and donors including any relevant peer‐reviewed literature that was missed in the systematic search. We iteratively revised the extraction and analysis plan as we reviewed the literature.

### 2.3 Eligibility criteria

We included original, peer‐reviewed research studies, published clinical or programme reports, and grey literature in English. These studies evaluated linkage to prevention and care using seven delivery approaches, namely case management, streamlined, partner services, incentives, virtual, peer‐based and QI interventions, reflecting discussions at the WHO on the most appropriate delivery approaches (Table 1). We excluded studies not written in English and those that did not meet the inclusion criteria based on study dates and intervention strategy.

**Table 1:** Definitions of delivery approaches and rationale for inclusion^a^

Approach	Definition	Rationale for inclusion in review
Streamlined interventions	Interventions that reduce the time between diagnosis and engagement in HIV prevention and care. Include any combination of: same‐day HIV testing, HIV prevention, antiretroviral therapy initiation, point‐of‐care CD4 cell count or viral load testing [3]	Introduced in 2018 WHO guidance on “test and start.” Included to identify new evidence and use in prevention context (e.g. same‐day PrEP initiation)
Case management	Client‐centred, multi‐step processes to coordinate access to medical and social supports to resources, including HIV‐specific services [20]	Introduced in 2018 WHO guidance and updated in 2019. Included to identify new evidence and use in prevention context.
Incentives	Financial or non‐financial rewards for linking to HIV care or prevention [21]	Previously reviewed in 2019 WHO guidance and addressed use in a good practice statement. Included to update evidence review of care and prevention.
Virtual interventions	Interventions that include social media, videos, text messaging, phone calls and computerized platforms [3]	Previously reviewed in 2019 and 2021 WHO guidance and addressed use in a good practice statement. Included to update evidence review of care and prevention.
Peer‐based	Interventions that enlist specific populations to support others to engage in services and adopt healthy lifestyles [22]	Previously reviewed in 2018 and 2019 WHO guidance. Included to update evidence review of care and prevention.
Partner services	Involves partner notification, contact tracing, index testing and family‐based index case testing for reaching the partners of people living with HIV and includes network‐based testing approaches [13]	Previously reviewed in 2019 WHO guidance. Included to update evidence review of care and prevention.
Quality improvement	Solutions to prevent errors and defects during HIV service delivery, for example score cards, plan‐do‐study act cycles [3]	Previously reviewed in 2018 WHO guidance. Included to update evidence review of care and prevention.
Multiple interventions	Combinations of more than one delivery approach mentioned above	Included to account for studies evaluating more than one intervention.

^a^ Support for disclosure was evaluated under partner services. Demand creation was excluded from this review due to the shift in focus at the WHO towards targeted HIV Test and Start programmes.

### 2.4 Outcomes

The main outcomes of interest were: (1) linkage to prevention—defined as connecting HIV‐negative individuals to relevant post‐test services [3]; (2) linkage to care—defined as accessing care at an HIV clinic after a positive HIV diagnosis measured either by the first clinic attendance date, first CD4 cell count, viral load date [23]; or ART initiation—defined as starting ART after an HIV‐positive result [3]. Costs associated with linkage to prevention and care were also of interest.

### 2.5 Data extraction

Two authors (PC and BW) were involved in literature selection and data extraction. Covidence^TM^ was used to upload data from the search databases before the removal of duplicate citations and article extraction onto an Excel spreadsheet. The extraction sheet contained citation elements (title, year of publication), location (country, WHO region), study design, results and size estimates where applicable. Costing information was also included where applicable. We adjusted costs to 2021 United States Dollars (USD$) using the Gross Domestic Product deflator [24]. Disagreements on the inclusion or exclusion of literature were resolved by consensus, and, if that failed, a third independent reviewer (CJ) resolved the disagreement.

### 2.6 Analysis

Data from the extraction sheet were first categorized into linkage to prevention and care then by delivery approach and study design, before being used to develop the manuscript. Qualitative data summarizing findings on the delivery approaches were grouped thematically.

## 3 RESULTS

### 3.1 Overview of results

#### 3.1.1 Search results

**Figure 1:**
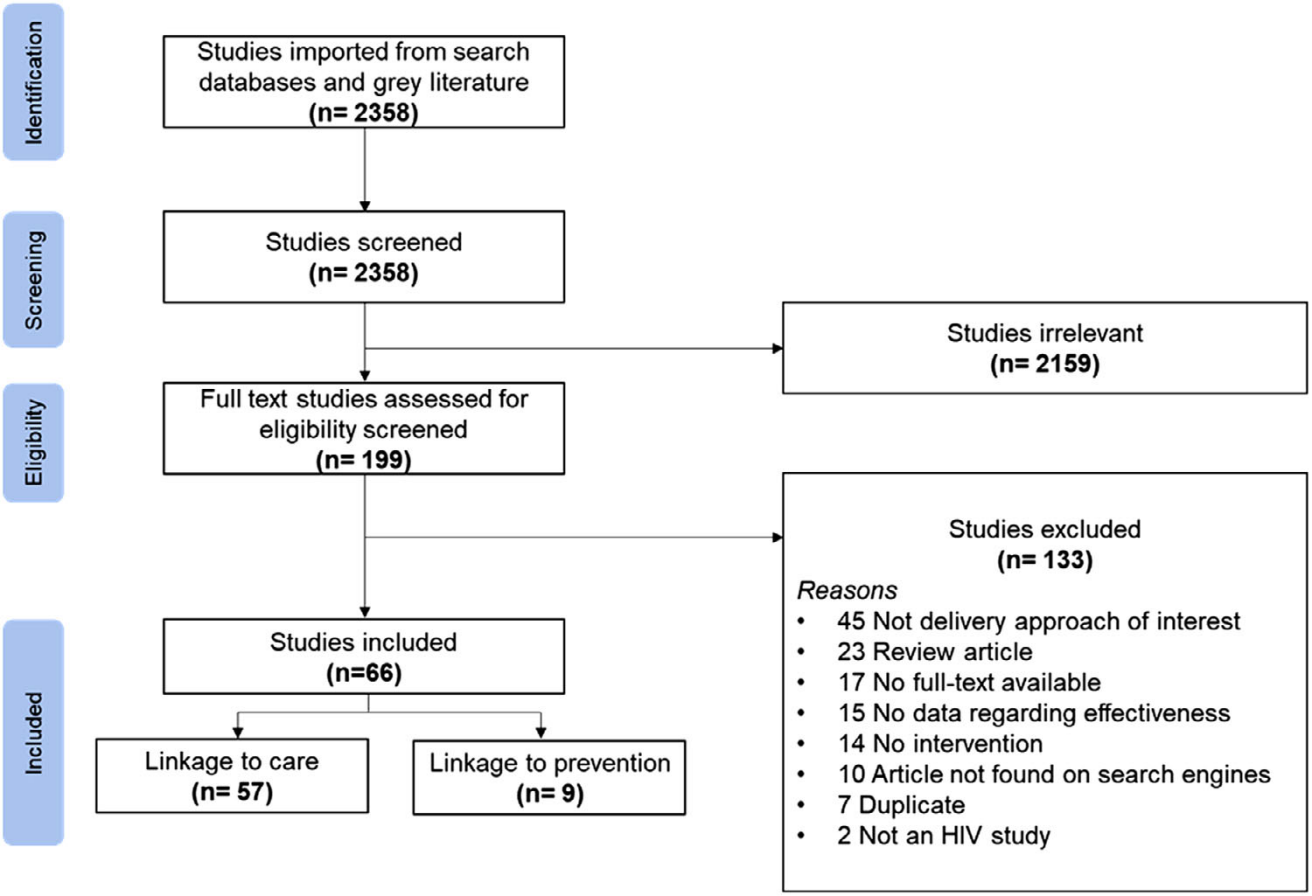
PRISMA flowchart for article selection.

Figure 1 indicates the search results and study selection process [25]. The search strategy yielded 2358 citations (including 12 grey literature) of which 199 full‐text articles were assessed for eligibility. Of these, 66 articles met our inclusion criteria: nine (14%) on linkage to prevention and 57 (86%) on linkage to care (Tables 2, 3, 4, 5, 6, 7).

**Table 2:** Summary of linkage to HIV prevention studies

Study	Population, type of testing	Delivery approach	Description	Results
**RCTs**				
Hewett et al. 2016 [26] Zambia RCT	Adults 18+ Facility‐based testing	Peer‐based	Intervention: Three arms Arm 1 (standard of care, SOC): Standard model of service provision at the entry point Arm 2: Enhanced counselling and referral to add‐on service with follow‐up Arm 3: Components of study arm two, with the additional offer of an escort	Voluntary medical male circumcision (VMMC) linkage: At 6 weeks, enhanced referral and escort increased VMMC when compared to standard of care (Relative risk, RR: 2.82, 95% CI: 1.60−4.98). Cervical cancer screening (CCS) linkage: At 6 weeks, cervical cancer screening increased with enhanced referral and escort when compared to standard of care (RR: 7.50, 95% CI: 4.77–11.78).
Schneider et al. 2021 [27] United States RCT	Adults 18−35 y MSM Facility‐based testing	Peer‐based	Intervention: Had two parts: (1) a half‐day, small group training workshop and (2) a series of check‐in calls (or “boosters”) Standard of care: Participants attended a sexual risk assessment workshop	Pre‐exposure prophylaxis (PrEP) referral: Over a 12‐month period, participants in the intervention group were more likely to be connected to PrEP referral or linkage services (adjusted odd's ratio, aOR 1.50 [1.09–2.06]; *p* = 0.01) compared to the control group.
Choko et al. 2019 [28] Malawi RCT	Adults 18+ Combination testing: Facility, HIVST, Community testing	Multiple interventions (incentives, virtual)	Five intervention arms: Letter and clinic access together with two prequalified oral HIV self‐test (HIVST) kits for the woman to take home for her male partner (“ST”). Conditional fixed cash financial incentive of $3 (“ST + $3”) Conditional fixed cash financial incentive of r $10 (“ST + $10”) 10% chance of winning $30 (“ST + lottery”). Included a phone call to the male partner on the day the woman enrolled (“ST + reminder”). Standard of care: Clinic invitation letter to the male partner	VMMC referral: Compared to SOC, the HIV self‐test (ST) + $3 and the ST + $10 interventions were associated with improved referral for VMMC with adjusted relative risk, aRR 3.76 (95% CI 1.76–8.03).
Barnabas et al. 2016 [29] Uganda, South Africa RCT	Adults 16+ Community‐based testing	Multiple interventions (streamlined, virtual, case management)	Intervention: Point‐of‐care (POC) group with same‐day point‐of‐care CD4 cell count testing at enrolment. HIV‐negative uncircumcised men (aged 16–49 years) who could receive secure mobile phone text messages were randomly assigned (1:1:1) to receive text message reminders, lay counsellor visits or standard clinic referral	VMMC linkage: 62 (28%) of 224 men were circumcised in the male circumcision clinic referral group compared with 137 (48%) of 284 men in the text message reminder group (relative risk 1.72, 95% CI 1.36–2.17) and 106 (47%) of 226 men in the lay counsellor follow‐up group (RR = 1.67, 1.29–2.14)
Wray et al. 2018 [30] United States RCT	Adults 18+ Men who have sex with men (MSM) Self‐testing	Virtual interventions	Intervention: eTest‐HIVST kits equipped with Bluetooth low‐energy (BLE) beacons were mailed to participant home addresses at 3‐month intervals. HIV test counsellors then called participants who opened their test kits within 24 hours to answer any questions and offer referrals to other sexual health services. Standard of care: HIV self‐test kits without beacons	Pre‐exposure prophylaxis (PrEP) referral: PrEP referral differed significantly between arms, *F* (2,62) = 3.44, *p* = 0.038, with eTest more likely to be referred for PrEP (m0−m2 = −0.24, SE = 0.12, *p* = 0.04)
Doblecki‐Lewis et al. 2019 [31] United States RCT	Adults 18+ MSM transgender women (TG) Facility‐based testing	Case management	Intervention: Strengths‐based case management with in‐person session with the patient navigator lasting 45−60 minutes Standard of care: Information package on HIV prevention strategies	PrEP initiation: 40% initiated PrEP within 12 weeks in intervention versus 29% in control (*p* value 0.37)
Joseph Davey et al. 2022 [32] South Africa RCT	Adults 18+ Self‐testing	Partner services	Intervention: Secondary distribution of HIVST by women living with HIV (WLHIV) to their male partners, that is index partner HIVST Standard of care (SOC): Invitation to facility‐based testing	PrEP initiation: Fewer male partners initiated PrEP within 3 months of study enrolment in the HIVST versus SOC arm (5% vs. 16%, RR = 0.44, 95% CI: 0.14–1.40, *p* = 0.27).
**Non‐RCT**				
Stankevitz et al. 2021 [33] Zimbabwe Mixed methods	Adults 15+ No testing/not reported	Case management	Intervention: “Test and Prevent” pilot programme involved risk assessment of all clients with a negative result from HIV testing (with national risk assessment tool), accompanied referral, fast‐tracking and targeting follow‐up. Standard of care: None	PrEP initiation: Among clients referred for PrEP (*n* = 206), 98% completed their referrals and started PrEP.
Storholm et al. 2021 [34] United States Quasi‐experimental	Adults 18+ MSM PWID Facility‐based testing	Streamlined interventions	Intervention: A 7‐item standardized post‐screening consultation and PrEP referral instrument was developed and integrated into the workflow of busy primary care clinics to help facilitate PrEP uptake among at‐risk men based on the 2017 CDC clinical practice guidelines assessed at post‐intervention Standard of care: 12 months preceding the intervention	PrEP referral: Significant increase in PrEP referrals overall during the screening intervention period as compared to the preceding 12 months. Change in referral rate from pre‐ to post‐interruption β2 (0.37, *p* = 0.01). Change in slope from pre‐ to post‐interruption β3 (−0.06, *p* = 0.00); Post interruption slope β1+β3 (−0.03, *p* = 0.04).
Mark et al. 2019 [35] Kenya Cohort	Adults 18+ Community‐based testing	Partner services	Intervention: Home‐based partner education and testing including sexually transmitted infection (STI) symptom recognition and treatment referral, engagement in HIV care and treatment services, and VMMC for HIV prevention among HIV‐negative men. Standard of care: Clinic invitation letter	STI consultations: Men who received the intervention were more likely to report an STI consultation (*n* = 47 vs. 16; RR = 1.59; 95% CI = 1.33−1.89). VMMC linkage: Few men of either arm sought circumcision: 3 (4%) of 72 of intervention men and 2 (2%) of 88 of control men reported obtaining circumcision (RR, 1.29; 95% CI, 0.62–2.70).

Abbreviation: HIVST, HIV self‐testing; MSM, men who have sex with men; PWID, persons who inject drugs; RCT, Randomized controlled trial

**Table 3:** Summary of linkage to HIV care studies

Study	Population, type of testing	Delivery approach	Description	Results
**RCTs**				
Desai et al. 2017 [36] Kenya RCT	Adults, adolescents and children Community‐based testing	Streamlined interventions	Intervention: Point‐of‐care CD4 cell count testing and additional counselling on the result by trial staff, including their ART eligibility on the basis of CD4 criteria at that time. Standard of care: CD4 cell counts done at one of three referral laboratories serving the study catchment area.	Linkage to care: Of 371 participants in the point‐of‐care group, 215 (58%) had linked to care within 6 months versus 108 (34%) of 321 in the standard‐of‐care group (Cox proportional multivariable hazard ratio [HR] 2.14, 95% CI 1.67–2.74; log rank *p*<0.0001)
Hayes et al. 2017 [37] Zambia RCT	Adults 18−44 y Community‐based testing	Streamlined interventions	Intervention: PopART—All community members offered including annual HTS, linkage to care services, VMMC, PMTCT, STIs and TB screening. Standard of care: No comparison arm; focused on intervention communities	Antiretroviral (ART) initiation: Proportion of known HIV‐positive individuals on ART increased from 54% to 74% in men and from 53% to 73% in women. The proportion of HIV‐positive adults on ART increased from 44% to 61%.
Labhardt et al. 2018 [7] Lesotho RCT	Adults 18+ Community‐based testing	Streamlined interventions	Intervention: Same‐day ART initiation after pre‐ART counselling. If they agreed to start therapy within the upcoming days, the study nurse left a 30‐day supply of ART. Standard of care group: Post‐test counselling and an appointment at their nearest health facility within the next 28 days.	Linkage to care: Linkage to care within 90 days after enrolment was 68.6% (94 of 137) in the same‐day group versus 43.1% (59 of 137) in the usual care group (absolute difference, 25.6%; 95% CI, 13.8−36.3%; *p* < 0.001)
Wu et al. 2017 [38] China RCT	Adults 18+ Facility‐based testing	Streamlined interventions	Intervention: One4All—Rapid, point‐of‐care HIV EIA screening and CD4 testing, with in‐parallel viral load testing to facilitate the completeness of diagnostic assessment Standard of care: Routine facility‐based care services	ART initiation: One4All group participants had increased odds of 90‐day ART initiation compared with standard‐of‐care group participants (OR 3.49, 95% CI 1.37–8.86, *p* = 0.01)
Aliyu et al. 2016 [39] Nigeria RCT	Adult women 16+ Facility‐based testing	Streamlined interventions	Intervention: Task shifting through the transition of decentralized PMTCT tasks to trained midwives, point‐of‐care CD4 testing, integrated mother and infant service provision, and male partner and community engagement. Standard of care: Health information, opt‐out HIV testing, infant feeding counselling, referral for CD4 cell counts and treatment, home‐based services, antiretroviral prophylaxis, and early infant diagnosis.	ART initiation: Mothers in the intervention group were more likely to initiate ART than mothers in the control group (adjusted relative risk [RR] 3.3, 95% CI 1.4–7.8).
Killam et al. 2010 [40] Zambia RCT stepped‐wedge design	Adult pregnant women 18+ Facility‐based testing	Streamlined interventions	Intervention: Women receiving integrated ART and ANC where ART services were provided 1–2 days per week. Standard of care: Women who started ANC more than 60 days before the intervention rollout	Linkage to care: A greater proportion enrolled while pregnant and within the 60 days of HIV diagnosis in the intervention cohort (376/846, 44.4%) compared with the control cohort (181/716, 25.3%), AOR 2.06, 95% CI (1.27–3.34) ART initiation: A greater proportion initiated ART while pregnant in the intervention cohort (278/846, 32.9%) compared with the control cohort (103/716, 14.4%), AOR 2.01, 95% CI (1.37–2.95).
Turan et al. 2015 [41] Kenya RCT	Adult pregnant women 18+ Facility‐based testing	Streamlined interventions	Intervention: ANC nurses at intervention sites received training on appropriate prescription and management of patients on HAART, while HIV‐positive women were offered enrolment and receipt of all HIV services at the ANC clinic, including HAART if eligible. Standard of care: Routine PMTCT services and referral of HIV‐positive pregnant women to a separate HIV clinic at the same facility.	Linkage to care: HIV care enrolment was higher in intervention compared to control clinics (69% vs. 36%, OR = 3.94, 95% CI: 1.14–13.63). ART initiation: Eligible women in the intervention arm were more likely to initiate ART (40% vs. 17%, OR = 3.22, 95% CI: 1.81–5.72).
Labhardt et al. 2014 [42] Lesotho RCT	Adults, adolescents and children Combination testing: Home based and mobile clinic	Streamlined interventions	Intervention: Mobile clinic (MC) Home‐based testing and counselling (HTC) group: Community campaigns with subsequent service provision in mobile clinics. Home‐based HTC group: Informed about community campaign 1 week prior to the scheduled date (though no community gathering, or health talk was held) and door‐to‐door visits	Linkage to care: Ten (25.6%) out of the 39 in the home‐based HTC group and 19 (25.3%) out of the 75 in the Mobile clinic HTC group linked to HIV care at the nearest facility within 1 month—adjusted OR 0.99, 95% CI (0.35‐2.79). *p* value 0.98
McNaghten et al. 2015 [43] South Africa, Uganda, Tanzania RCT	Adults 18−49 y Facility‐based testing	Streamlined interventions	Intervention: HIV testing was provided using three different models, after clinical consultation (model A), during clinical consultation (model B) and before clinical consultation (model C) Standard of care: None	Linkage to care: The percentage of referred patients who entered care was highest for the after clinical consultation—model A (74.4%) compared with during clinical consultation—model B (54.8%) and before clinical consultation—model C (55.6%); though not statistically significant
Gardner et al. 2016 [44] United States RCT	Adults 18+ PWID No testing/not reported	Case management	Intervention: Anti‐Retroviral Treatment and Access to Services (ARTAS) intervention used strengths‐based case management with a linkage coordinator to support linkage Standard of care: Passive referral following counselling	Linkage to care: Drug users reporting methadone maintenance became engaged in care in less than half the time of drug users without a treatment history (HR 2.97 [1.20, 6.21]).
Neduzhko et al. 2020 [45] Ukraine RCT	Adults 18+ Facility‐based testing	Case management	Intervention: Participants were introduced to the Linkage Coordinator (LC)—a trained nurse who provided the MARTAS (Modified ARTAS) where ARTAS was an individual‐level, multi‐session case management linkage‐to‐care intervention, based on the Strengths‐based Case Management (SBCM) model Standard of care: Verbal referrals to a network of government AIDS Centres	Linkage to care: The intervention was associated with higher likelihood of linkage to HIV care (84.4% vs. 33.8%; crude RR 2.50; 95% CI 1.96, 3.19; *p* < 0.001; adjusted RR 2.45; 95% CI 1.72, 3.47; *p*<0.001)
Ruzagira et al. 2017 [46] Uganda RCT	Adults 18+ Community‐based testing	Case management	Intervention: Home‐based HIV counselling and testing (HBHCT) with referral and counselling at 1 and 2 months Standard of care: HBHCT and referral only	Linkage to care: One hundred and twenty‐seven (42.1%) participants linked to care: 76 (51.0%) in the intervention arm versus 51 (33.3%) in the control arm (odds ratio = 2.18, 95% confidence interval [CI] = 1.26–3.78; *p* = 0.01).
Samet et al. 2019 [47] Russia RCT	Adults 18−70 y PWID No testing/not reported	Case management	Intervention: Strengths‐based case management. Peer case managers where delivered the intervention via five one‐on‐one sessions over a 6‐month period. Standard of care: Resource card containing harm reduction information and contact information for HIV care	Linkage to care: At 6 months post‐enrolment, 51% of the intervention group and 31% of controls linked to HIV care (adjusted odds ratio [AOR] 2.34; 95% confidence interval [CI]: 1.49, 3.67; *p* < 0.001).
Surratt et al. 2014 [48] United States RCT	Adults 18−50 y FSW PWID Facility‐based testing	Case management	Intervention: Strength‐Based Case Management (SBCM) plus a professional peer. Standard of care: SBCM only	Linkage to care: Strength‐Based Case Management, SBCM only (Cohen's *d*: −0.69, 95% CI [−1.96, 0.22]), SBCM plus peer (Cohen's *d*: −0.38, 95% CI [−1.39, 0.47])
Gordon et al. 2018 [49] United States RCT	Adults 18+ Community‐based testing	Case management	Intervention: Project Bridge (PB): Case managers and social workers were assigned to provide intensive case management and weekly sessions to participants for 3 months with follow up at 6 and 12 months post randomization. Standard of care: Standard referral to treatment.	Linkage to care: No statistically significant difference in participant initiation in community HIV treatment by treatment condition (χ^2^ (1) = 0.079, *p* = 0.78). ART initiation: Of the 99 participants who were interviewed at 3‐month follow‐up, 69% entered HIV treatment (Project Bridge PB, men = 26/38, women = 7/11 vs. Standard of care, men = 28/39, women = 7/11, χ^2^ (1) = 0.333, *p* = 0.56).
Wanyenze et al. 2011 [50] Uganda RCT	Adults 18+ Facility‐based testing	Case management	Intervention: Inpatient HIV counselling and testing (HCT), that is personalized risk assessment and risk reduction plan. In both intervention and control groups, HIV‐positive individuals were given referrals to HIV/AIDS clinics for follow‐up care Standard of care: Outpatient HCT 1‐week post‐discharge	Linkage to care: 73.6% (39) in the control attended an HIV clinic compared to 55.8% (53) in the intervention arm (χ^2^ = 4.58, *p* = 0.03)
Elul et al. 2017 [51] Mozambique RCT	Adults 18+ Facility‐based testing	Multiple interventions (streamlined, virtual, incentives)	Intervention: Combination intervention strategy (CIS) included (1) real‐time, point‐of‐care CD4 test results; (2) patients with CD4 cell count < = 350 cells/mm^3^ were provided with accelerated ART initiation; (3) health messages and appointment reminders via SMS; and (4) patients in the CIS‐positive cohort received the CIS plus non‐cash financial incentives (Fis), that is prepaid cellular air‐time cards Standard of care: Managed as per prevailing Ministry of Health guidelines	Linkage to care: 57% of participants in the CIS group achieved the primary outcome versus 35% of those in the standard of care, SOC group (RR CIS vs. SOC = 1.58, 95% CI 1.05–2.39).
McNairy et al. 2017 [52] Eswatini RCT	Adults 18+ No testing/not reported	Multiple interventions (streamlined, virtual, incentives)	Intervention: CIS, that is point‐of‐care CD4^+^ testing at the time of an HIV‐positive test, accelerated antiretroviral therapy (ART) initiation for treatment‐eligible participants, mobile phone appointment reminders, health educational packages and non‐cash financial incentives. Standard of care: Post‐test counselling and referral to an HIV clinic using a national referral form	Linkage to care: 705 (64%) participants at sites randomized to the CIS study arm and 477 (43%) participants at sites randomized to the SOC study arm achieved linkage to HIV care within 1 month of HIV‐positive testing plus retention in HIV care at 12 months after HIV testing. Accounting for clustering within study units, the RR was 1.52 (95% CI 1.19–1.96, *p* < 0.01)
Barnabas et al. 2020 [53] South Africa RCT	Adults 18+ Facility‐based testing	Multiple interventions (virtual, incentives)	Intervention: Conditional lottery incentive group plus motivational text messages (SMS + Lottery) Standard of care: Motivational text messages (SMS) only.	ART initiation: Compared to motivational text messages, lottery incentives decreased the median time to ART initiation from 126 to 66 days (*p* = 0.0043, age‐adjusted Cox regression) among all participants, and, from 134 to 20 days (*p* = 0.0077) among participants who were not virally suppressed at baseline.
Barnabas et al. 2016 [29] Uganda, South Africa RCT	Adults 16+ Community‐based testing	Multiple interventions (streamlined, case management)	Intervention: Point‐of‐care group with same‐day point‐of‐care CD4 cell count testing at enrolmentEligible HIV‐positive participants (aged ≥16 years) were randomly assigned (1:1:1) to receive Lay counsellor follow‐up arm: Participants received visits at home at months 1, 3 and 6. Clinic facilitation arm: A lay counsellor met HIV‐positive people at the clinic and explained the steps of engagement in care and benefits of ART or Standard of care (Referral arm): Referral to local HIV clinics to obtain their count.	ART initiation: ART initiation was higher in the lay counsellor follow‐up arm (185/449, 41%) than in the referral arm (142/423, 34%) (RR = 1.23, 95% CI 1.02, 1.47). ART initiation in the clinic facilitation arm was not clearly higher (161/431, 37%) compared to the referral arm (RR = 1.11, 95% CI 0.92, 1.34).
Hoffmann et al. 2017 [54] South Africa RCT	Adults 18+ Community‐based testing	Multiple interventions (streamlined, incentives, case management)	Intervention arms: Arm 1‐Point of care CD4 testing with printed results given to participant within 20 minutes Arm 2‐Point of care CD4 testing plus five strengths‐based counselling sessions (care facilitation) Arm 3‐Point of care CD4 testing plus transport reimbursement of US$ 6 for urban and peri‐urban participants and US$ 10 for rural participants. Standard of care: Counselling and referral letter to a convenient HIV care clinic	Linkage to care: POC CD4 testing plus care facilitation increased linkage to care from 29% (standard of care) to 38%, *p* value 0.001. Other arms did not show effectiveness.
Solomon et al. 2014 [55] India RCT	Adults 18+ PWID No testing/not reported	Incentives	Intervention: Incentives (INC) arm: Participants were given voucher incentives (range $4−$8) for achieving specific targets, for example ART initiation, visits to ART centre, in addition to receiving a referral letter to a government ART centre Standard of care: Referral letter to a government ART centre	Linkage to care: Significantly more participants from the intervention arm visited a government ART centre (49 vs. 33; *p* < 0.01). ART initiation: Twenty‐seven (45%) participants in the incentives arm and 16 participants (26.7%) in the control arm initiated ART (hazard ratio = 2.33 [95% confidence interval, 1.15–4.73]).
El‐Sadr et al. 2017 [56] United States RCT	Adults 18+ No testing/not reported	Incentives	Intervention: Individuals who tested positive for HIV at a financial incentives (FI) HIV test site received a coupon redeemable within 3 months for two cash equivalent gift cards: $25 on getting blood drawn for HIV‐related tests and $100 on meeting with a clinician and developing a care plan. Standard of care: No financial incentives	Linkage to care: Financial incentives did not significantly increase linkage to care compared with SOC with (aOR 1.10, 95% CI: 0.73−1.67; *p* = 0.65)
Maughan‐Brown et al. 2018 [57] South Africa RCT	Adults 18+ Community‐based testing	Incentives	Intervention: Voucher that could be exchanged for R300 cash (∼$23) if ART was started within 3 months. Standard of care: Follow‐up telephone counselling by the mobile clinic staff to encourage linkage to care.	Linkage to care: No significant differences were found between the incentive and non‐incentive group in terms of linkage to care (adjusted odds ratio [aOR]: 0.70, 95% confidence interval [CI]: 0.26–1.91) ART initiation: No significant differences in initiation of ART (aOR: 0.67, 95% CI: 0.26–1.78).
Ayieko et al. 2018 [58] Kenya RCT	Adults 15+ Community‐based testing	Virtual interventions	Intervention: Clinical officers, trained on patient‐centred care, made calls to participants exploring barriers to treatment and describing options for spaced clinic visits, flexible clinic hours, appointment reminders and so on. Standard of care: Standard HIV counselling	Linkage to care: Intervention group participants were more likely to link to care within 7 days compared with those in the control group (24/68 [35%] vs. 12/62 [19%]; *p* = 0.043). The effect of the intervention was maintained at 30 days (28/68 [41%] vs. 15/62 [24%]; *p* = 0.04).
Chang et al. 2021 [59] Uganda RCT	Adults 15+ No testing/not reported	Virtual interventions	Intervention: CHWs (“health scouts”) were trained and subsequently delivered theory‐based situated motivational interviewing counselling that was guided by an algorithm in a mobile phone application, to promote engagement in HIV treatment and prevention services. Standard of care: Health Scouts were instructed not to conduct mobile health‐supported counselling home visits with residents in the control clusters.	Linkage to care: There was a higher HIV care coverage among HIV‐positive participants in the intervention compared with the control arm (Prevalence risk ratio, PRR: 1.06, 95% CI: 1.01−1.10, *p* = 0.01). ART initiation: There was higher ART coverage (PRR: 1.05, 95% CI: 1.01−1.10, *p* = 0.03) among HIV‐positive participants in the intervention compared with the control arm.
Kuo et al. 2019 [60] United States RCT	Adults 18+ Prison No testing/not reported	Virtual interventions	Intervention: CARE+ Corrections tool with computerized counselling session plus post‐incarceration text messaging intervention. An interactive, audio‐narrated computerized motivational interview that assessed a person's risk, HIV care behaviours and provided an individualized risk and linkage to care Standard of care: Educational video on opioid overdose prevention and printout of local HIV providers and resources.	Linkage to care: No significant differences between those in the CARE+ Corrections arm versus the control arm (AOR 1.18; 95% CI 0.25‐5.53)
Chanda et al. 2017 [61] Zambia RCT	Adults 18+ FSW Self‐testing	Peer‐based	Arm 1: Direct delivery of the HIV self‐test from the peer educator to the participant (henceforth, delivery) Arm 2: Distribution of a coupon from the peer educator to the participant that could be used for collection of an HIV self‐test from a fixed distribution point (coupon). Arm 3 (standard of care): Referral to standard testing.	Linkage to care: Three‐quarters (74.6%) of participants in the standard‐of‐care arm reported they had sought care following their positive test, compared to 51.0% in the delivery arm and 52.8% in the coupon arm. (*p*‐value comparing arm 1 with standard of care 0.13 and comparing arm 2 with standard of care 0.17)
Gengiah et al. 2021 [62] South Africa RCT	Adults 18+ Facility‐based testing	Quality improvement	Intervention: Quality improvement with three key components: clinical and QI skills training, on‐site mentorship of nurse supervisors and clinic staff, and data quality improvement activities to enhance the accuracy and completeness of routine clinic data Standard of care: Monthly supervision and data feedback meetings.	ART initiation: The percentage of patients initiated on ART (91.7 vs. 95.5; RR = 0.96 [0.90–1.02]; *p* = 0.17) was similar in both groups.
Pearson et al. 2014 [63] United States RCT	Adults, 18+ Prisoner Facility‐based testing	Quality improvement	Intervention: Quality improvement strategies through Network for the Improvement of Addiction Treatment (NIATx) trained coaches to help local agency change teams learn how to try out and assess new organizational processes for targeted improvements such as improved patient retention in treatment for offenders under correctional supervision. Standard of care: Staff received HIV training only	Linkage to care: NIATx did not increase linkage to treatment, log OR 0.70 (95% CI = –0.33, 1.74; *p* = 0.18)
Cherutich et al. 2017 [64] Kenya RCT	Adults 18+ Combination testing: Facility‐based, community‐based	Partner services	Intervention: Immediate arm—Health advisors (HAs) immediately traced sex partners to newly diagnosed HIV positive clients, offered HTS and referred to care. Standard of care: Delayed group—HAs did not provide any additional support to promote partner notification or testing until 6 weeks after the participant's enrolment.	Linkage to care: Those newly enrolled to care, that is immediate group 88/586 (15%) versus delayed group 19/680 (3%), (IRR [95% CI] 4.4 [2.6−7.4])
Joseph Davey et al. 2022 [32] South Africa RCT	Adults 18+ Self‐testing	Partner services	Intervention: Secondary distribution of oral HIV self‐test kits by women living with HIV (WLHIV) to their male partners, that is index partner HIVST Standard of care (SOC): Invitations for men's facility‐based testing	ART initiation: In the HIVST arm, fewer male partners initiated ART within 3 months of study enrolment as compared to SOC (67% vs. 100%, RR = 0.67, 95% CI: 0.42–1.06, *p* = 0.37)
**Non‐RCT**				
Tattersall et al. 2022 [65] Canada Cross‐sectional	Adults 19+ Facility‐based testing	Case management	Intervention: Seek and Treat for Optimal Prevention of HIV/AIDS (STOP HIV/AIDS) offered provincial level training of service providers, routine offers of HIV testing in all healthcare settings, and provided assistance for outreach nursing teams to support engagement and retention on ART. 1 January 2010−31 December 2018 was the post‐intervention cohort. Standard of care: 1 January 2000−31 December 2009 was the pre‐intervention cohort	ART initiation: STOP HIV/AIDS era participants were 5.97 times more likely to initiate ART than pre‐STOP HIV/AIDS participants (adjusted hazards ratio [aHR] 5.96, 95% CI 4.47–7.97; *p* < 0.001).
Kenya 2014 [66] United States Cross‐sectional	Adults 18+ PWID No testing/not reported	Case management	Intervention: Having a case manager for HIV‐positive PWID Standard of care: Not having a case manager	ART initiation: Participants who had a case manager at the time of the study interview were significantly more likely to have been referred to case management when first diagnosed with HIV, take HIV medications (16.8% vs. 44.7%), and had received HIV care within the past 6 months of the study interview
Camacho‐Gonzalez et al. 2017 [67] United States Cohort	Adults 18–24 y Community‐based testing	Case management	Intervention: Youth diagnosed with HIV at non‐traditional venues and received motivational interviewing, and case management. Standard of care: Youth seen at an adolescent HIV clinic	Linkage to care: Linkage was higher in the intervention arm (88%) compared to standard of care (39%), *p* value <0.001
Kuhns et al. 2021 [68] United States Cohort	Adults 18+ TG No testing/not reported	Case management	Intervention: TransLife Care (TLC) “one stop shop” provided “bundled” housing, employment, legal and outreach‐based health services (i.e. triage, health education and referral), in addition to HIV case management. Standard of care: Up to 6 months prior to intervention	Linkage to care: Receipt of the intervention (vs. none), was associated with any HIV care visit (aOR 2.05; 95% CI 1.25–3.37; *p* = 0.005), more total HIV care visits (aRR 1.45; 95% CI 1.09–1.94; *p* = 0.011), being retained in care (aOR 1.58; 95% CI 1.03–2.44; *p* = 0.038) and having a viral load test done (aOR 1.95; 95% CI 1.23–3.09; *p* < 0.01).
Herce et al. 2018 [69] Zambia Quasi‐experimental	Adults 18+ Facility‐based testing	Streamlined interventions	Intervention: Had five core components: (1) health worker training and mentorship; (2) timely provider‐initiated HIV testing and counselling (PITC); (3) on‐site HIV care enrolment; (4) dedicated ART clinic days; and (5) synchronized TB and HIV patient follow‐up. Analysed as post‐intervention cohort (1 August 2011−31 March 2012) Standard of care: Pre‐intervention cohort (1 June 2010−31 January 2011)	Linkage to care: By 12 weeks after anti‐TB therapy (ATT) initiation, 62.9% of patients in the post‐intervention cohort had linked to HIV care, compared to 54.7% in the pre‐intervention cohort (*p* = 0.03).
McKay et al. 2021 [70] United States Others Simulated Environment	Adolescents, adults 13–24 y Facility‐based testing	Streamlined interventions	Two simulated interventions: Expanding testing sites Using current testing sites but improving direct referral to linkage to care LTC staff from organizations providing testing Standard of care: None	Linkage to care: Improving direct referral to the linkage to care (LTC) programme decreased days to successful linkage from an average of 30 to 23 days but expanding testing sites increased average days to 31 days unless those sites also made direct referrals.
Garofalo et al. 2022 [71] Nigeria Others Non randomized controlled trial	Adolescents, adults 15–24 y MSM Combination testing: Facility, community or home‐based testing	Multiple interventions (peer‐based, virtual)	Intervention: Four peer navigators conducted social media outreach promoting sexual health and guiding individuals to HIV counselling and rapid testing in clinical, community or home‐based settings. Standard of care: Historical HIV testing surveillance data among all men aged 15−24 years at catchment clinics	Linkage to care: 339 participants underwent testing for HIV. Thirty‐six participants (10.6%) were confirmed as HIV seropositive and 31 (86.1%) were linked to care.
Brantley et al. 2019 [72] United States Cross‐sectional	Adults 18+ Prison No testing/not reported	Multiple interventions (case management, virtual)	Intervention: Re‐entry services were provided on‐site by the corrections specialist, that is education on services in their community that they may qualify for, referral to medical care, assistance making the first HIV medical appointment, assistance with enrolment. Persons were then offered video conferencing intervention if they requested a referral to case management Standard of care: Clients who consented to enrolment but were ultimately unable to participate in a video conference in time for their release due to scheduling conflicts	Linkage to care: No statistically significant difference in linkage to care rate between the intervention and comparison groups (AOR = 1.2; 95% CI 0.6–2.3, *p* > 0.05)
Hacking et al. 2019 [73] South Africa Mixed methods	Adolescents, adults 12−25 y Facility‐based testing	Multiple interventions (peer‐based, virtual)	Intervention: Peer support and virtual mentorship where patients were added to a mentee sign‐up sheet, which included their name, contact details and preferred platform of communication (e.g. WhatsApp and SMS). Mentees were then assigned to appropriate mentors matched based on similar demographics or circumstances. Standard of care: Two matched controls who tested positive at the same facility.	Linkage to care: Linkage to care at any point after diagnosis was substantially higher in the virtual mentees’ cohort (80% vs. 43%).
MacKellar et al. 2018 [74] Tanzania Others Programme evaluation	Adults, adolescents and children Combination testing: Facility‐based, community‐based	Multiple interventions (peer‐based, virtual, case management)	Intervention: Recommended package of linkage services that included peer‐delivered linkage case management and treatment navigation, face‐to‐face counselling and telephone support, and retraining in linkage case management practices. Standard of care: Had no control group	Linkage to care: During three periods with different ART‐eligibility thresholds (CD4<350 [Oct 2014–Dec 2015, *n* = 2233], CD4 < 500 [Jan 2016–Sept 2016, *n* = 1221] and Test & Start [Oct 2016–May 2017, *n* = 752]), 90%, 96% and 97% of clients enrolled in HIV care ART initiation: During three periods with different ART‐eligibility thresholds, 47%, 67% and 86% of clients initiated ART, respectively, within 3 months of diagnosis.
Ruria et al. 2017 [75] Kenya Quasi‐experimental	Adolescents, adults 15–21 y No testing/not reported	Peer‐based	Intervention: Fast‐track peer‐navigated services, peer counselling, and psychosocial support at healthcare facilities and schools assessed postintervention, that is July 2016–December 2016 Standard of care: 6‐month period from July 2015 to December 2015	Linkage to care: The proportion of adolescent and youth clients who were linked to care increased from 56.5 pre‐intervention to 97.3% post‐intervention (*p* < 0.001)
Yan et al. 2014 [76] China Cross‐sectional	Adults 18+ MSM Community‐based testing	Peer‐based	Intervention: MSM peer‐led rapid HIV testing programme through mobile and community‐based organizations to reach previously untested MSM, link to care those confirmed HIV positive, and ascertain retention in care at 6 months. Standard of care: HIV serological surveillance surveys of the MSM population between April and June 2012	Linkage to care: Higher proportions of HIV‐positive MSM screened received their confirmatory test results (98.1% vs. 72.6%, *p*<0.001) and linked to care (90.4% vs. 42.0%, *p*<0.001) compared to standard of care
O'Laughlin et al. 2021 [77] Uganda Cohort	Adults 18+ Facility‐based testing	Virtual interventions	Intervention: Weekly phone calls for 12 weeks from a research assistant trained in HTS. If the client was not reached initially, a maximum of three attempted calls were made per week. Additionally, literate patients received weekly SMS reminders for 12 weeks. Standard of care: Individuals unwilling or ineligible to participate	Linkage to care: Excluding those who linked prior to receipt of intervention, the intervention improved linkage (69 [68%] vs. 50 [47%], *p* < 0.01).
Phanuphak et al. 2020 [78] Thailand Cohort	Adults 18+ MSM TG Combination testing: Self‐testing; facility‐based	Virtual interventions	Intervention: Enrolled participants were allowed to self‐select from three HIV counselling and testing strategies: (1) offline HIV counselling and testing (Offline group); (2) online pre‐test counselling and offline HIV testing (Mixed group); and (3) online counselling and online, supervised, HIV self‐testing (Online group). Followed up at 6, 12 months to ascertain ART initiation. Standard of care: None	ART initiation: Among 60 baseline HIV positive and 18 seroconversion participants, successful ART initiation in the Online group (52.8%) was lower than the Offline (84.8%) and Mixed groups (77.8%).
Brizzi et al. 2020 [79] United States Quasi‐experimental	Adults 18+ No testing/not reported	Quality improvement	Intervention: ARV stewardship involved the HIV PharmD reviewing the electronic medical record (EMR) for each patient daily (Monday through Friday), ensuring that ART and/or OI medications were ordered and administered during the inpatient setting. Transitions of care (TOC) component involved the HIV PharmD ensuring that patients had access to medications at the time of hospital discharge. Evaluated at the post‐implementation phase (1 July 2018 and 31 December 2018) Standard of care: Pre‐implementation phase (1 July 2017 and 31 December 2017)	Linkage to care: Linkage to care rates increased significantly from 78% (46/59) to 92% (61/66) in the intervention arm (RR, 1.19; 95% CI, 1.03–1.42; *p* = 0.02)
Boeke et al. 2018 [80] Uganda Quasi‐experimental	Adults 18+ No testing/not reported	Quality improvement	Intervention: Training of expert patients and facility staff, and provision of financial and logistical support of facilities. Evaluated 9 months after implementation Standard of care: 9 months before implementation of the intervention	Linkage to care: Linkage rates at 3 months in the intervention compared to the standard of care was not statistically significant (OR 1.10, 95% CI: 0.77−1.57, *p* value 0.60)
Mark et al. 2019 [35] Kenya Cohort	Adults 18+ Community‐based testing	Partner services	Intervention: Home‐based partner education and testing including STI symptom recognition and treatment referral, engagement in HIV care and treatment services, and VMMC for HIV prevention among HIV‐negative men. Standard of care: Clinic invitation letter	Linkage to care: A small proportion of newly diagnosed HIV‐positive male participants reported linking to care during the course of the study: 4 of 15 (27%) in the intervention arm and 3 of 5 (60%) in the control arm (RR, 0.66; 95% CI, 0.34–1.29).

Abbreviation: HIVST, HIV self‐testing; MSM, men who have sex with men; PMTCT, prevention of mother to child transmission; PWID, persons who inject drugs; RCT, Randomized controlled trial; TB, tuberculosis

**Table 4:** Summary results from RCTs indicating effect sizes (*n* = 27)

Study	Population, type of testing	Delivery approach	Description	Result type	Effect type	Effect size	Lower bound 95% CI	Upper bound 95% CI	Positive Non‐significant Negative 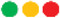
**Linkage to prevention**									
Hewett et al. 2016 [26] Zambia RCT	Adults 18+ Facility‐based testing	Peer‐based	Standard service provision at the entry point (arm 1); enhanced counselling and referral with follow‐up (arm 2); arm two plus additional offer of an escort (arm 3)	Voluntary medical male circumcision (VMMC) linkage Cervical cancer screening (CCS)	RR (VMMC: Enhanced referral + escort)	2.82	1.6	4.98	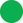
					RR (VMMC: Enhanced referral only)	1.97	1.09	3.58	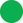
					RR (VMMC: SOC)	Ref			
					RR (CCS: Enhanced referral + escort)	7.5	4.77	11.78	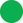
					RR (CCS: Enhanced referral only)	6.22	3.95	9.80	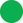
					RR (CCS: SOC)	Ref			
Schneider et al. 2021 [27] United States RCT	Adults 18–35 y MSM Facility‐based testing	Peer‐based	Peer‐based check‐in calls with participants	PrEP referral	OR	1.5	1.09	2.06	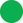
Choko et al. 2019 [28] Malawi RCT	Adults 18+ Combination testing: Facility, HIVST, Community testing	Multiple interventions (incentives, virtual)	Five intervention arms. Arm 1 HIVST kits for the woman to take home for her male partner (“ST”). Arms 2, 3 Two arms offered an additional conditional fixed cash financial incentive of $3 or $10 (“ST + $3” and “ST + $10,” respectively). Arm 4 Offered a 10% chance of winning $30 (“ST + lottery”). Arm 5 Included a phone call to the male partner on the day the woman enrolled (“ST + reminder”).	VMMC or ART referral	aRR (ST + $10)	3.76	1.76	8.03	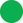
					aRR (ST + $3)	3.06	1.43	6.57	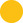
					aRR (ST + lottery)	1.87	0.58	6.01	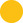
					aRR (ST + reminder)	1.68	0.90	3.15	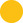
					aRR (ST only)	1.41	0.79	2.50	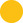
					aRR (SOC)	Ref			
**Linkage to care**									
Gardner et al. 2016 [44] United States RCT	Adults 18+ PWID No testing/not reported	Case management	Strengths‐based case management with a linkage coordinator	Linkage to care	HR	2.97	1.2	6.21	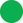
Neduzhko et al. 2020 [45] Ukraine RCT	Adults 18+ Facility‐based testing	Case management	Strengths‐based case management with a linkage coordinator	Linkage to care	RR	2.45	1.72	3.47	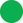
Ruzagira et al. 2017 [46] Uganda RCT	Adults 18+ Community‐based testing	Case management	Counselling support within case management	Linkage to care	OR	2.18	1.26	3.78	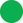
Samet et al. 2019 [47] Russia RCT	Adults 18−70 y PWID No testing/not reported	Case management	Strengths‐based case management	Linkage to care	aOR	2.31	1.49	3.67	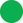
Surratt et al. 2014 [48] United States RCT	Adults 18−50 y FSW PWID Facility‐based testing	Case management	Strengths‐based case management	Linkage to care	Cohen's *d*	−0.38	−1.39	0.47	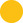
Aliyu et al. 2016 [39] Nigeria RCT	Adult women 16+ Facility‐based testing	Streamlined interventions	PMTCT task‐shifting to trained midwives, POC CD4 testing, male partner and community engagement	ART initiation	RR	3.3	1.4	7.8	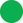
Desai et al. 2017 [36] Kenya RCT	Adults, adolescents and children Community‐based testing	Streamlined interventions	POC CD4 testing, assessment of ART eligibility	Linkage to care	HR	2.14	1.67	2.74	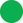
Killam et al. 2010 [40] Zambia RCT stepped‐wedge design	Adult pregnant women 18+ Facility‐based testing	Streamlined interventions	Integration of ART in ANC	Linkage to care	aOR	2.06	1.27	3.34	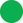
				ART initiation	aOR	2.01	1.37	2.95	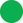
Labhardt et al. 2014 Lesotho RCT	Adults, adolescents and children Combination testing: Home based and Mobile clinic	Streamlined interventions	Home‐based HTS compared with mobile clinic HTS involving health talks at participating villages	Linkage to care	aOR	0.99	0.35	2.79	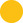
Labhardt et al. 2018 [7] Lesotho RCT	Adults 18+ Community‐based testing	Streamlined interventions	Same‐day ART initiation after pre‐ART counselling	Linkage to care	Absolute difference	0.26	0.14	0.36	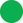
Turan et al. 2015 [41] Kenya RCT	Adult pregnant women 18+ Facility‐based testing	Streamlined interventions	ART initiation at PMTCT through task‐shifting to ANC nurses	Linkage to care ART initiation	OR OR	3.94 3.22	1.14 1.81	13.63 5.72	
Wu et al. 2017 [38] China RCT	Adults 18+ Facility‐based testing	Streamlined interventions	POC enzyme immunoassay (EIA) and CD4 testing with parallel viral load testing	ART initiation	OR	3.49	1.37	8.86	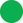
Chang et al. 2021 [59] Uganda RCT	Adults 15+ No testing/not reported	Virtual interventions	Mobile phone application enabled motivational interviewing and counselling	Linkage to care ART initiation	PRR PRR	1.06 1.05	1.01 1.01	1.1 1.10	
Kuo et al. 2019 [60] United States RCT	Adults 18+ Prison No testing/not reported	Virtual interventions	Interactive, audio‐narrated computerized motivational interview	Linkage to care	aOR	1.18	0.25	5.53	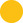
El‐Sadr et al. 2017 [56] United States RCT	Adults 18+ No testing/not reported	Incentives	$25 gift card upon blood draw for CD4 count and viral load tests, and $100 gift card upon meeting with a clinician to develop a care plan	Linkage to care	aOR	1.1	0.73	1.67	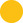
Maughan‐Brown et al. 2018 [57] South Africa RCT	Adults 18+ Community‐based testing	Incentives	$25 cash voucher for ART initiation within 3 months	Linkage to care ART initiation	aOR aOR	0.7 0.67	0.26 0.26	1.91 1.78	
Solomon et al. 2014 [55] India RCT	Adults 18+ PWID No testing/not reported	Incentives	$4−$8 vouchers for linking to care and ART initiation	ART initiation	HR	2.33	1.15	4.73	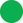
Gengiah et al. 2021 [62] South Africa RCT	Adults 18+ Facility‐based testing	Quality improvement	Training and mentorship of staff in clinical skills and data quality improvement	ART initiation	RR	0.96	0.9	1.02	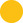
Pearson et al. 2014 [63] United States RCT	Adults, 18+ Prisoner Facility‐based testing	Quality improvement	Coaching of staff in targeted processes, for example improving patient retention, for offenders under correctional supervision	Linkage to care	Log OR	0.7	−0.33	1.74	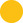
Cherutich et al. 2017 [64] Kenya RCT	Adults 18+ Combination testing: Facility‐based, community‐based	Partner services	Immediate tracing, HTS and linkage to care for sex partners to newly diagnosed HIV‐positive clients	Linkage to care	IRR	4.4	2.6	7.4	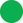
Joseph Davey et al. 2022 [32] South Africa RCT	Adults 18+ Self‐testing	Partner services	Secondary distribution of oral HIV self‐test kits by women living with HIV (WLHIV) to their male partners	ART initiation PrEP initiation	RR RR	0.67 0.44	0.42 0.14	1.06 1.40	
Barnabas et al. 2016 [29] Uganda, South Africa RCT	Adults 16+	Multiple interventions (streamlined, virtual)	POC CD4 testing with either lay counsellor follow‐up, clinic facilitation or standard referral for HIV‐positive clients, or text message reminders, lay counsellor visits, or standard clinic referral for HIV‐negative uncircumcised men	ART initiation	RR (Lay counsellor)	1.23	1.02	1.47	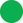
	Community‐based testing				RR (Clinic facilitation)	1.11	0.92	1.34	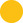
					Ref: Standard of care	Ref			
					RR (Text message)	1.72	1.36	2.17	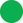
					RR (Lay counsellor)	1.67	1.29	2.14	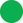
					Ref: Standard clinic referral	Ref			
Elul et al. 2017 [51] Mozambique RCT	Adults 18+ Facility‐based testing	Multiple interventions (streamlined, incentives, virtual)	Combined POC CD4 testing, accelerated ART initiation, text reminders and non‐financial cash incentives (prepaid airtime cards)	Linkage to care	RR	1.58	1.05	2.39	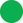
McNairy et al. 2017 [52] Eswatini RCT	Adults 18+ No testing/not reported	Multiple interventions (streamlined, incentives, virtual)	Combined POC CD4 testing, accelerated ART initiation, text reminders and non‐financial cash incentives (prepaid airtime cards)	Linkage to care	RR	1.52	1.19	1.96	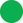

Abbreviation: HIVST, HIV self‐testing; MSM, men who have sex with men; PWID, persons who inject drugs; PMTCT, prevention of mother to child transmission; RCT, Randomized controlled trial

**Table 5:** Summary of costing/cost‐effectiveness analysis articles

Study	Population, type of testing	Intervention	Delivery approach	Description	Results (adjusted to USD 2021)
Choko et al. 2019 [28] Malawi RCT	Adults 18+ Combination testing: Facility, HIVST, Community testing	Linkage to prevention	Multiple interventions (incentives, virtual)	Five intervention arms: Letter and clinic access together with two prequalified oral HIVST kits for the woman to take home for her male partner (“ST”). Conditional fixed cash financial incentive of $3 (“ST + $3”) Conditional fixed cash financial incentive of $10 (“ST + $10”) 10% chance of winning $30 (“ST + lottery”). Included a phone call to the male partner on the day the woman enrolled (“ST + reminder”). Standard of care: Clinic invitation letter to the male partner Costing: All resources were costed and used to estimate the total costs. Total costs were divided by the total number of men who tested for HIV and attended a male‐friendly clinic (MFC) to estimate the cost per male partner who tested for HIV and attended an MFC, and by the total number of men who started ART or were referred for VMMC to estimate the cost per male partner who tested for HIV and either started ART or was referred for VMMC. 2016 USD	The average cost per male partner who started ART or was referred for VMMC for the five intervention arms ranged from $106 (ST + $3) to $189 (ST + lottery).
Hewett et al. 2016 [26] Zambia RCT	Adults 18+ Facility‐based testing	Linkage to prevention	Peer‐based	Intervention: Three arms Arm 1 (standard of care): Standard model of service provision at the entry point Arm 2: Enhanced counselling and referral to add‐on service with follow‐up Arm 3: Components of study arm two, with the additional offer of an escort Cost‐effectiveness analysis: Discounted DALY measures and projected lifetime treatment costs were used to calculate incremental cost‐effectiveness ratios 2014 USD	Compared to SOC, arms 2 and 3 had incremental cost per DALY averted for VMMC at $433 and $186, respectively, and for cervical cancer screening at $697 and $122, respectively
Sharma et al. 2016 [81] Kenya RCT	Adult pregnant women 18+ Community‐based testing	Linkage to prevention	Partner services	HOPE intervention: Couples received a home visit in which study staff (health advisors) screened male partners and offered couples HIV counselling and testing. Standard of care: Written invitation to bring their partners to the clinic for couple HIV testing. Cost‐effectiveness analysis: Microcosting of the HOPE intervention (payer perspective) to estimate programme costs as well as a lower cost scenario of task‐shifting to community health workers. Parameterized a mathematical model of HIV transmission. 2014 USD	Incremental cost of adding the intervention to standard antenatal care was $36–42 and $16–18 per couple tested with programme and task‐shifting costs, respectively. The incremental cost‐effectiveness ratio (ICER) was $1017 and $706 per disability‐adjusted life year averted for the programme and task‐shifting scenarios, respectively.
Boeke et al. 2018 [80] Uganda Quasi‐experimental	Adults 18+ No testing/not reported	Linkage to care	Quality improvement	Intervention: Training of expert patients and facility staff, and provision of financial and logistical support of facilities. Evaluated 9 months after implementation Standard of care: 9 months before implementation of the intervention Costing: Programme costs, that is cost of trainings, facility funding for phone airtime and home visits, and programme supervision were used to estimate the cost per additional patient linked to care and cost per additional patient retained in care by dividing the cost to the number of additional patients linked to and retained in care over that timeframe. 2016 USD	The annual cost per additional patient retained in care was estimated to be $53.
MacKellar et al. 2018 [74] Tanzania Others Programme evaluation	Adults, adolescents and children Combination testing: Facility‐based, community‐based	Linkage to care	Multiple interventions (case management, virtual)	Intervention: Recommended package of linkage services that included peer‐delivered linkage case management and treatment navigation, face‐to‐face counselling and telephone support, and retraining in linkage case management practices. Standard of care: Had no control group Costing: Total and per‐client programme costs were estimated from data collected retrospectively and included personnel, travel, commodities, training, vehicles and equipment costs converted to 2017 USD	Estimated per‐client cost was $49 for delivering the package of linkage services in communities and facilities overall, and $20 USD for a facility‐only model with task shifting.
Masiano et al. 2023 [82] DRC RCT	Adult pregnant women 18+ Facility‐based testing	Linkage to care	Incentives	Intervention: PMTCT care plus $5 on the first visit which increased by $1 at every scheduled visit thereafter but was reset to $5 for missing a scheduled visit Standard of care: PMTCT care alone Cost‐effectiveness analysis: Calculated incremental cost‐effectiveness ratios by taking the product of incremental costs and incremental effectiveness. Included formal healthcare sector costs (e.g. drugs), informal costs (e.g. patient transport costs) and productivity losses captured using the opportunity cost of time spent seeking healthcare service. 2016 International $	The mean total cost per participant was $580 among women in the intervention group (PMTCT plus cash transfers) compared to $485 in the standard of care group (PMTCT alone) for an incremental cost of $96 (95% CI: 66, 125). The incremental cost‐effectiveness associated with the intervention was $669 (95% CI: 619, 718) for an additional woman taking PMTCT services and $1156 (95% CI: 1047, 1265) for an additional woman retained in PMTCT care, compared to the standard of care.
Sharma et al. 2017 [83] Kenya RCT	Adults 18+ Combination testing: Facility‐based, community‐based	Linkage to care	Partner services	Intervention: Immediate arm—Health advisors (HAs) immediately traced sex partners to newly diagnosed HIV positive clients, offered HTS and referred to care. Standard of care (Delayed group)—HAs did not provide any additional support to promote partner notification or testing until 6 weeks after the participant's enrolment. Cost‐effectiveness analysis: Individual‐based, dynamic HIV transmission model. Estimated costs for both a programme scenario and a task‐shifting scenario using community health workers. Simulated 200 cohorts of 500,000 individuals 2014 USD	The incremental cost effectiveness ratio of immediate aPS was $1255 and $956 per disability‐adjusted life year averted under the programme and task‐shifting scenario, respectively, when compared to the delayed arm.
Ulrich et al. 2022 [84] Peru Others Multi‐phase	Adults 18+ MSM TG Community‐based testing	Linkage to care	Multiple interventions (streamlined, virtual)	Intervention: Combination intervention strategy (CIS): Point‐of‐care CD4^+^ testing at the time of an HIV‐positive test, accelerated antiretroviral therapy (ART) initiation for treatment‐eligible participants, mobile phone appointment reminders, health educational package Standard of care: Annual serologic testing Cost‐effectiveness analysis: Compartmental model of HIV transmission to evaluate the cost‐effectiveness of Sabes compared to SOC using a government healthcare perspective, 20‐year time horizon and 3% annual discounting. Estimated the proportion of cases of HIV detected during early primary infection, reduction in HIV incidence and prevalence, incremental cost‐effectiveness ratio (ICER) and net monetary benefit. 2017 USD	Sabes had an ICER of $1580 per QALY compared to SOC. Intervention costs were $7613 per early primary infection diagnosed.

Abbreviation: HIVST, HIV self‐testing; MSM, men who have sex with men; PMTCT, prevention of mother to child transmission; RCT, Randomized controlled trial

**Table 6:** Summary of values, preferences and gaps from the qualitative studies

	Key findings	Gaps
**Linkage to HIV prevention**		
Case management [33]	Additional support for accompanied referrals and fast‐tracking encouraged clients to access PrEP	Some providers did not complete screening if they perceived clients to be at low risk for HIV, or if they had a heavy workload
**Linkage to HIV care**		
Incentives [85, 86, 87]	Cash incentives were appreciated. Other options to access healthcare were available, for example walking, or borrowing money to go to facilities	Cash incentives introduce moralizing discussions on who can be influenced and what can be bought
Case management [88]	Patients saw the case manager/linkage coordinator as an accessible healthcare specialist helping them navigate complicated healthcare systems.	Lack of psychological support from health providers was a key challenge
Virtual [73, 89]	Mobile phone app to facilitate HIVST showed great potential Virtual platforms using texts (WhatsApp and SMS) to support peer mentorship were appreciated by adolescents and young adults	Participants preferred models allowing some degree of direct human interaction, for example face‐to‐face or through phone calls Concerns about data privacy, for example possibility of phone hacking
Peer‐based [90]	Peer‐led community‐based HIV self‐testing improved access to HIV testing	Concerns about privacy and confidentiality of HIV test results.

HIVST, HIV self‐testing.

**Table 7:** Gaps in WHO recommendations and implementation guidance

Intervention	Delivery approach	WHO recommendation and implementation guidance
Linkage to HIV prevention	Streamlined interventions	None
	Case management	None
	Incentives	None
	Virtual interventions	✓
	Peer‐based	✓
	Partner services	✓
	Quality improvement	None
Linkage to HIV care	Streamlined interventions	✓✓
	Case management	✓✓
	Incentives	✓✓
	Virtual interventions	✓✓
	Peer‐based	✓✓
	Partner services	✓✓
	Quality improvement	✓✓

✓✓, recommendations and implementation guidance; ✓, implementation guidance.

#### 3.1.2 Geographic distribution of the studies

**Figure 2:**
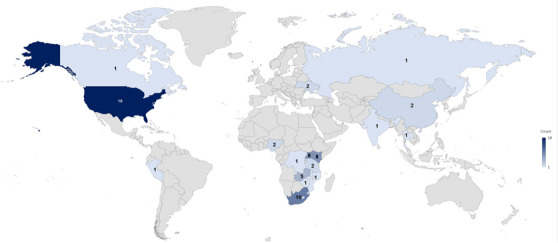
Map of studies included in scoping review, by country. In Africa, Lesotho (*n* = 2) and Eswatini (*n* = 1) are too small to appear on the map. The nine linkage to prevention studies were conducted in the United States [4] and in Africa (Kenya [*n* = 2], Zambia, Malawi and Zimbabwe).

Figure 2 represents the geographic distribution of the studies with none reported from the Eastern Mediterranean region. Among the nine linkage to prevention studies, five were from Africa and four were from the United States. Among the linkage to care studies, 60% were conducted in Africa (*n* = 34/57) and 28% in the Americas (*n* = 16/57).

#### 3.1.3 Study designs

**Figure 3:**
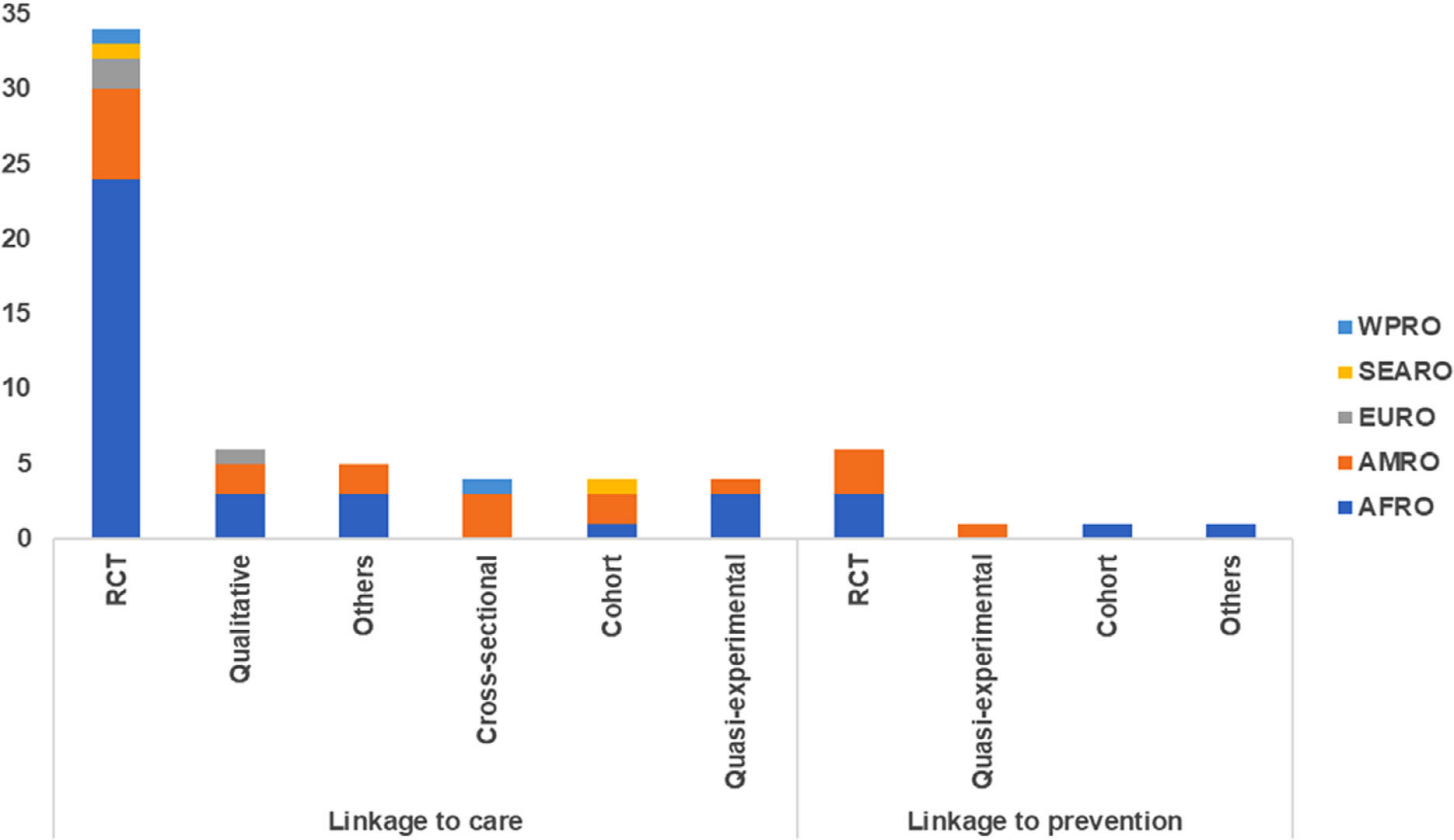
Type of intervention by study design and WHO region. AFRO, WHO Regional Office to Africa; AMRO, WHO Regional Office to the Americas; EMRO, WHO Regional Office to the Eastern Mediterranean; EURO, WHO Regional Office to Europe; RCT, randomized controlled trial; SEARO, WHO Regional Office to South‐east Asia; WPRO, WHO Regional Office to the Western Pacific.

Of the nine linkage to prevention studies, six (67%) were randomized controlled trials (RCTs) (Figure 3). Of the 57 linkage to care studies, 34 were RCTs (60%), six were qualitative (11%), five comprised of other study designs, for example programme evaluations (9%), four were cross‐sectional (7%), four were cohort (7%) and four were quasi‐experimental (7%) studies.

#### 3.1.4 Delivery approaches

**Figure 4:**
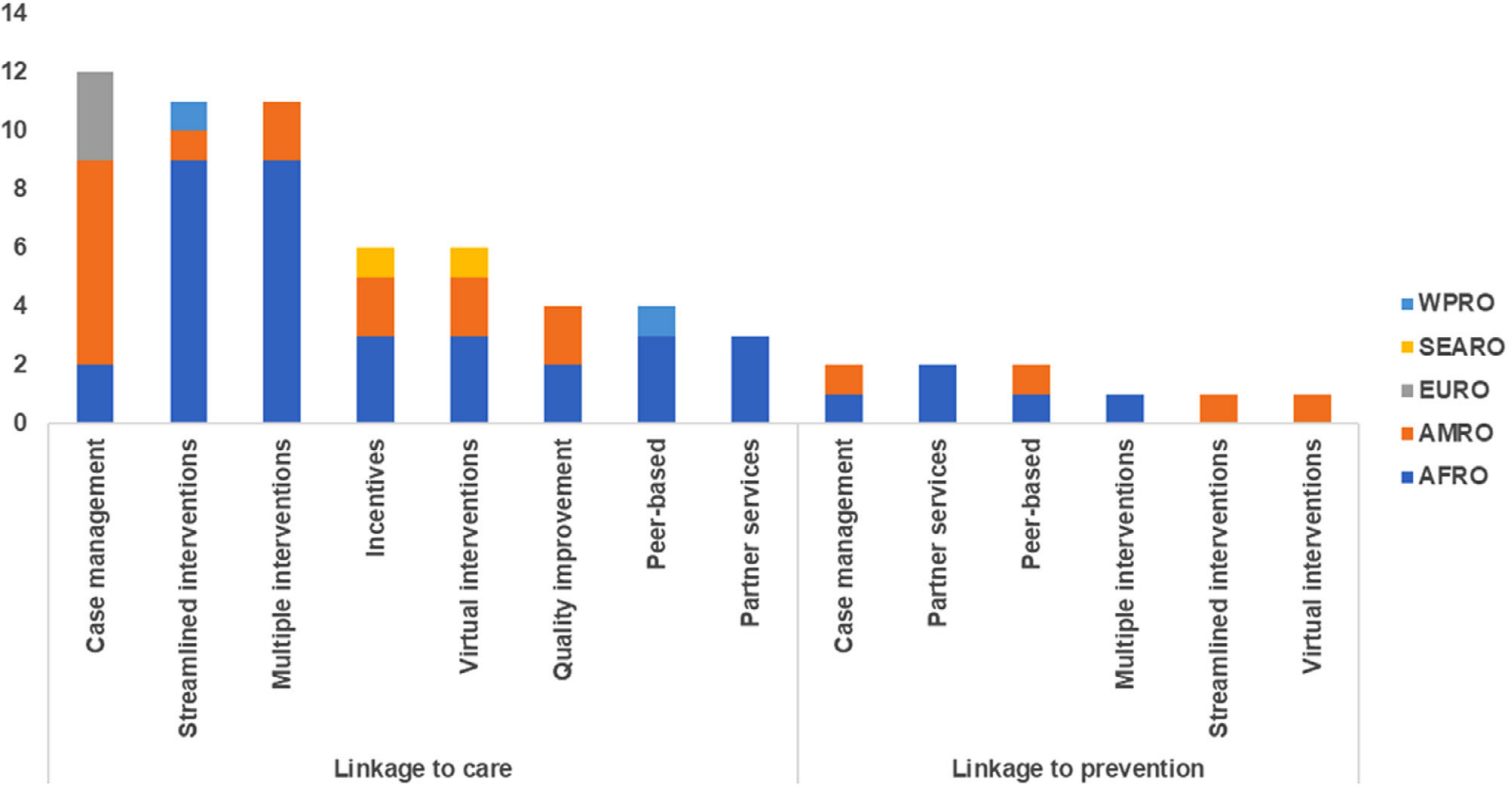
Type of intervention by delivery approach and WHO region. AFRO, WHO Regional Office to Africa; AMRO, WHO Regional Office to the Americas; EMRO, WHO Regional Office to the Eastern Mediterranean; EURO, WHO Regional Office to Europe; SEARO, WHO Regional Office to South‐east Asia; WPRO, WHO Regional Office to the Western Pacific.

Among linkage to prevention studies, case management, partner services and peer‐based models were the most commonly used delivery approaches (each at 22%, *n* = 2/9) (Figure 4). For linkage to care studies, case management (21%, *n* = 12/57) was most commonly described especially in the Americas, followed by streamlined and multiple interventions (each at 19%, *n* = 11/57), especially in Africa.

#### 3.1.5 HTS approaches

**Figure 5:**
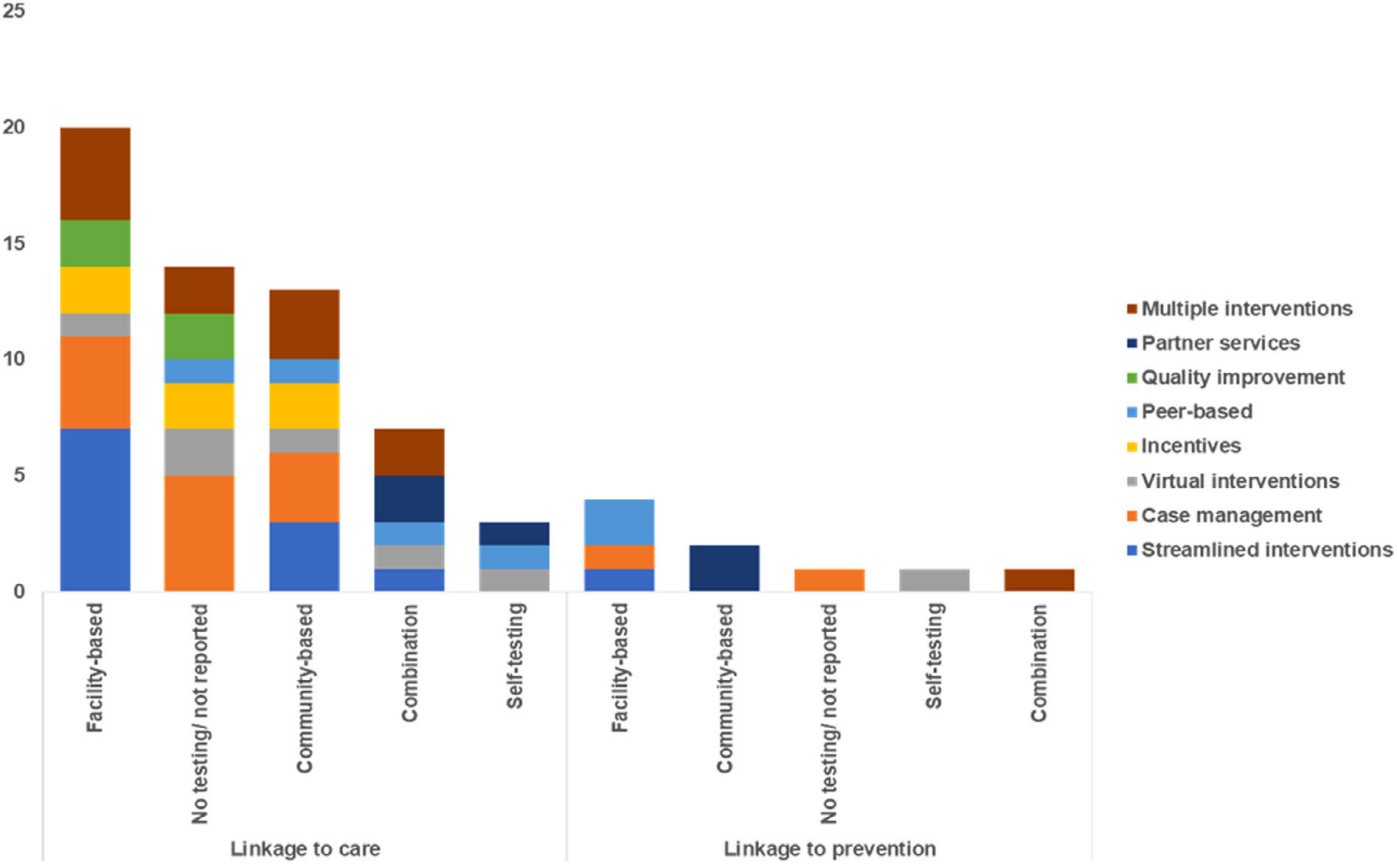
HIV testing strategy by intervention and delivery approach.

HTS approaches varied across studies (Figure 5). Among linkage to prevention studies, most utilized facility‐based HTS (44%, *n* = 4/9), especially for peer‐based interventions. Community‐based HTS (22%, *n* = 2/9) was primarily used to deliver partner services.

Among the linkage to care studies, facility‐based HTS was commonly used (35%, *n* = 20/57) to deliver streamlined interventions. HTS approaches were not reported (25%, *n* = 14/57), especially among case management studies. Community‐based HTS was particularly used (23%, *n* = 13/57) for studies delivering multiple interventions.

### 3.2 Linkage to prevention

Nine linkage to prevention studies were conducted in Africa and the United States (Tables 2 and 4). Gaps in WHO recommendations on linkage to prevention are summarized in Table 7. Studies conducted in Africa were all among heterosexual individuals and focused on linkage to PrEP, VMMC, STI and cervical cancer screening [26, 28, 29, 31, 33, 35]. US‐based studies evaluated PrEP linkage among key populations [27, 30, 32, 34]. No linkage to HIV prevention study reported on QI.

#### 3.2.1 RCTs

*Peer‐based*: Two RCTs evaluated peer‐based interventions (22%, *n* = 2/9). In a peer‐based RCT among heterosexual adults in Zambia, there was increased VMMC uptake and cervical cancer screening among participants who received an enhanced referral and additional escort relative to those receiving routine counselling [26]. In a peer‐based RCT conducted in the United States, men who have sex with men (MSM) who received a training workshop and a series of check‐in phone calls had improved PrEP initiation compared to those who only attended a sexual risk assessment workshop [27].

*Multiple interventions*: Two RCTs evaluated multiple interventions (22%, *n* = 2/9). In a Malawi‐based cluster RCT, male partners of female antenatal care (ANC) attendees who received invitation letters, HIV self‐testing (HIVST) and financial incentives had higher VMMC referrals compared to those who received invitation letters only [28]. In an RCT conducted in South Africa and Uganda, men receiving either text message reminders or lay counsellor follow‐up were more likely to be circumcised compared to those receiving standard clinic referrals [29].

*Virtual interventions*: One study (11%, *n* = 1/9), a US‐based RCT, showed improved PrEP initiation among MSM who received phone‐based counselling from providers within 24 hours of opening electronically tagged HIVST kits compared to those receiving HIVST without electronic beacons [30].

*Case management*: One study (11%, *n* = 1/9), a US‐based RCT, showed no improvement in PrEP initiation among MSM receiving strengths‐based case management compared to those receiving an information package on HIV prevention strategies [31].

*Partner services*: One study (11%, *n* = 1/9), a South Africa‐based RCT, had fewer male sex partners initiating PrEP after receiving HIVST kits from their female partners compared to those invited to the facility for HTS [32].

#### 3.2.2 Non‐RCT study designs

Three studies involved non‐RCT study designs (Tables 2 and 4). In a Zimbabwe‐based mixed methods in evaluating case management, 98% of participants with a negative HIV result who received risk assessment and targeted follow‐up completed their referrals and initiated PrEP [33]. A US‐based quasi‐experimental study showed increased PrEP referral in the post‐intervention period among MSM receiving streamlined service delivery after post‐screening risk assessment [34]. In a Kenya‐based cohort study that involved contacting male partners of female ANC attendees, there was no improvement in VMMC linkage, though STI consultations were higher compared to the standard of care [35].

#### 3.2.3 Cost and cost‐effectiveness

Three studies, all in Africa, evaluated the costs of linking to prevention (Table 5). In Malawi, the average cost per male partner initiating ART or referred for VMMC among male sex partners to female ANC attendees ranged from US$106 (HIVST + $3 cash incentive) to US$189 (HIVST + lottery with 10% chance of winning $3 cash incentive) [28]. In Zambia, the incremental cost‐effectiveness ratio (ICER) comparing enhanced counselling with follow‐up (arm two) and components of arm two with escort (arm three) to standard of care was $433 and $186 per disability adjusted life year (DALY) averted for linking to VMMC, respectively, and $697 and $ 122 per DALY averted for cervical cancer screening, respectively [26]. In Kenya, the ICER for couples receiving home visits for HTS compared to those receiving written invitations was $1017 and $706 per DALY averted for the programme and task‐shifting scenarios, respectively [81].

#### 3.2.4 Qualitative values and preferences

In a Zimbabwe‐based mixed methods study evaluating values and preferences among providers offering risk assessment and targeted follow‐up to participants with negative HIV results, some providers purposefully did not complete screening if they perceived their clients to be at low‐risk for HIV, or if they had a heavy workload [33] (Table 6).

### 3.3 Linkage to care

Of the 57 linkage to care studies reported, 60% were RCTs (*n* = 34/57) (Tables 3 and 4). Gaps in WHO recommendations on linkage to care are summarized in Table 7. Thirty‐four studies were conducted in Africa, 16 in the Americas (mainly the United States), three in Europe and two each from the Western Pacific and Southeast Asia regions (Figure 3).

#### 3.3.1 RCTs

*Streamlined interventions*: Nine RCTs evaluated streamlined interventions (16%, *n* = 9/57). Three involved either wide‐scale community‐based testing [7, 36, 37] or facility‐based HTS [38] with point‐of‐care (POC) CD4 cell count testing and encouraged same‐day ART initiation all showed improved linkage to care and ART initiation. Three studies among pregnant women and mothers attending integrated ANC that offered ART also showed increased linkage to ART initiation [39, 40, 41]. Two studies that showed no effect in linkage to care included: (1) a study comparing home‐based to mobile‐clinic‐based HTS [42]; and (2) a study evaluating HTS provided before, during or after clinical consultation [43].

*Case management*: Seven RCTs evaluated case management (12%, *n* = 7/57). Four RCTs utilizing case managers in the United States, Russia, Ukraine and Rwanda showed increased linkage to care when compared to passive referral [44, 45, 46, 47]. However, two US‐based RCTs, one involving strengths‐based case management among female sex workers (FSWs) and persons who inject drugs (PWIDs) [48], and the other involving intensive case management and weekly counselling sessions for 3 months [49] showed no change in linkage to care or ART initiation. In Uganda, hospitalized clients receiving inpatient HTS and case management with a personalized risk assessment and risk reduction plan were less likely to link to care compared to those receiving outpatient HTS 1 week post‐discharge [50].

*Multiple interventions*: Five RCTs combined incentives, streamlined, virtual and/or peer‐based interventions (9%, *n* = 5/57). Two RCTs in Mozambique and Eswatini that improved linkage to HIV care (RR > 1.5) utilized a combination intervention strategy, that is POC CD4 testing, accelerated ART initiation, mobile phone appointment reminders, health educational packages and non‐cash financial incentives [51, 52]. In Uganda and South Africa, participants receiving conditional lottery incentives with motivational text messages had a shorter median time to ART initiation compared to those who received text messages alone [53]. In two South Africa‐based RCTs, one evaluating combined POC CD4 testing and strengths‐based case management [54], and the other evaluating same‐day POC CD4 testing and text message reminders [29] showed improved linkage to ART initiation.

*Incentives*: Three RCTs evaluated the use of cash vouchers valued at approximately $25 (range: $4−$100) to improve linkage to care (5%, *n* = 3/57). One India‐based RCT among PWIDs showed higher linkage to care and ART initiation among those receiving relative to the standard of care [55]. The other two RCTs that showed no effect on linkage to care included a US‐based RCT among PLHIV who received cash vouchers for HIV‐related tests [56], and a South Africa‐based RCT where PLHIV received approximately $25 if ART was started within 3 months of HTS [57].

*Virtual interventions*: Three RCTs utilized either phone, text, social media or mobile phone applications (5%, *n* = 3/57). In Uganda, HIV‐positive clients receiving motivational interviewing and counselling community health workers (CHWs) guided by a mobile phone algorithm had higher linkage to care in HIV services compared to those counselled by CHWs not guided by the algorithm [59]. In Kenya, participants receiving patient‐centred counselling phone calls on ART initiation from clinical officers had a higher likelihood of linking to care compared to those receiving routine counselling [58]. However, in a US‐based RCT among prisoners anticipating release, there was no significant difference between those who utilized computerized counselling with post‐incarceration text messaging compared to those who received an instructional video pre‐discharge [60].

*Partner services*: Partner services were reported in two studies in Kenya and South Africa (4%, *n* = 2/57). Positive results were reported in a cluster‐RCT on assisted partner services in Kenya where sex partners who were immediately contacted for HTS by a healthcare worker had higher linkage to care compared to those who were contacted 6 weeks later [64]. However, there was no effect on linkage to care in South Africa where fewer male sex partners initiated ART after receiving HIVST kits from their female partners compared to those receiving facility invitations [32].

*Quality improvement*: Two cluster‐RCTs in the United States and South Africa showed no change in linkage to HIV care (4%, *n* = 2/57). In the United States, there was no difference in linkage to care when comparing facilities receiving HIV training and QI strategies for targeted improvements, to those that only offered HIV training [63]. In South Africa, there was no difference in linkage to care when comparing facilities incorporating on‐site staff mentorship and data QI activities to facilities that only offered monthly supervision and data feedback meetings [62].

*Peer‐based*: One Zambia‐based cluster RCT (2%, *n* = 1/57) among adult FSWs showed no effect on linkage to care when HIVST kits were delivered either directly by a peer educator or through a coupon when compared to standard HTS [61].

#### 3.3.2 Non‐RCTs

Linkage to care studies involving non‐RCT study designs are summarized in Table 3.

*Case management*: Four non‐RCT studies (7%, *n* = 4/57) evaluated case management interventions. In a Canada‐based cross‐sectional study offering outreach nursing teams to support ART initiation and retention, participants were six times more likely to initiate ART compared to those enrolled pre‐intervention [65]. A US‐based cross‐sectional study improved linkage to HIV care among PWID utilizing linkage coordinators compared to those who did not [66]. A US‐based cohort study among youth living with HIV receiving HIV case management at non‐traditional HIV testing venues (e.g. night clubs) showed improved linkage to HIV care compared to those seen at routine adolescent clinics [67]. In another US‐based cohort study, transgender women who received strengths‐based case management together with housing, employment and outreach‐based health services were almost twice as likely to initiate and be retained in care compared to those who enrolled in the pre‐intervention period [68].

*Multiple interventions*: Four non‐RCT studies (7%, *n* = 4/57) evaluated multiple interventions. In a Nigeria‐based non‐randomized controlled trial, 86% (*n* = 31/36) of young men, including MSM, who received social media enabled peer‐navigation support linked to HIV care [71]. There was no effect on linkage to HIV care in a US‐based cross‐sectional study among prisoners evaluating a combined intervention of video conferencing, case management, pre‐release and re‐entry services compared to those who did not receive the combined intervention [72]. In a South Africa‐based mixed‐methods study, adolescents and youth receiving virtual peer support and mentorship through WhatsApp and SMS were more likely to link to care compared to matched controls [73]. In a Tanzania‐based programme evaluation, 97% of participants receiving peer‐delivered linkage case management, treatment navigation and face‐to‐face counselling enrolled in HIV care [74].

*Streamlined interventions*: Two non‐RCT studies (4%, *n* = 2/57) evaluated streamlined interventions. One was a Zambia‐based quasi‐experimental study where patients receiving integrated ART and Tuberculosis (TB) care were more likely to link to care compared to controls [69]. The other was a US‐based simulation study that showed improved direct referral to linkage to care among adolescents and young adults using expanded testing sites [70].

*Peer‐based*: Two studies (4%, *n* = 2/57) evaluated peer‐based interventions, and both showed positive results. In a Kenya‐based quasi‐experimental study, peer support increased linkage to care among adolescents and youth receiving fast‐tracked peer‐navigated services and counselling at healthcare facilities and schools compared to those in the pre‐intervention period [75]. In a China‐based cross‐sectional study, peers to previously untested MSM were more likely to test and link to care after rapid HTS at mobile and community‐based organizations compared to those who did not receive the intervention [76].

*Virtual*: Two studies (4%, *n* = 2/57) evaluated virtual interventions. In a Uganda‐based cohort study, participants receiving weekly phone calls and SMS reminders were more likely to link to HIV care compared to those who did not receive the intervention [77]. In Thailand, a study comparing MSM and transgender women using either online, supervised or offline HIV counselling and testing, there was lower ART initiation among participants in the online only group (52.8%) compared to those who received either facility‐based HTS (offline, 84.8%) or online pre‐test counselling with offline HIV testing using HIVST (mixed group, 77.8%) [78].

*Quality improvement*: Two studies (4%, *n* = 2/57) evaluated QI interventions. In a US‐based quasi‐experimental study, there was improved linkage to care after the implementation of a QI intervention in which pharmacists ensured ART and/or medications for opportunistic infections were ordered and administered in inpatient settings and that patients had access to medications at the time of hospital discharge compared to the pre‐implementation phase [79]. In a Uganda‐based quasi‐experimental study, there was improved linkage to HIV care in facilities where staff and expert patients were trained on QI compared to the pre‐intervention period [80].

*Partner services*: One study (2%, *n* = 1/57) evaluated interventions which contacted partners. In a Kenya‐based cohort study, there was no difference in linkage to care when comparing men contacted by their female partners who had attended ANC to those who received a clinic invitation letter [35].

#### 3.3.3 Cost and cost‐effectiveness

Five linkage to care studies reported on cost (9%, *n* = 5/57) (Table 5). In Uganda where staff and expert patients were trained on QI, the annual cost per additional patient retained in care was estimated at US $53 [80]. In Tanzania, a package of linkage services that included peer‐delivered case management and treatment navigation was estimated to cost US $49 per‐client in communities and facility settings overall, and US $20 for a facility‐only model where tasks were shifted from nurses to expert‐client counsellors [74]. In Kenya, the ICER for partner services was $1255 and $956 per DALY averted under programme and task‐shifting scenarios, respectively [83]. In the Democratic Republic of Congo, the ICER associated with $5 incentives for linking to prevention of mother to child transmission (PMTCT) was $669 and $1156 per additional woman taking PMTCT services and retained in PMTCT care, respectively, compared to standard of care [82]. In Peru, the ICER associated with rapidly linking treatment‐eligible PLHIV to CD4 cell count testing and ART compared to standard of care was $1580 per quality adjusted life year [84].

#### 3.3.4 Qualitative values and preferences

Seven linkage to care studies reported qualitative results (12%, *n* = 7/57) (Table 6). In two studies on cash‐based incentives (∼$25) in the United States and South Africa, participants felt that while incentives may increase linkage to care, they may also elicit ethical discussions [86, 87].

Two studies from South Africa reported qualitative results from multiple interventions. One evaluating integrated POC CD4 cell count testing, transport support and care facilitation reported improved psychosocial support from care facilitation that promoted engagement in care [85]. However, though transport cost reimbursement was appreciated by many participants, it was not the only means for managing transport costs for those who could either walk to the clinic or borrow money to pay for transport. The other, evaluating virtual peer mentorship (WhatsApp and SMS) among adolescents and youth, showed that even though mentees valued virtual mentorship, they preferred hybrid models allowing some degree of direct human interaction [73].

Three studies separately reported on qualitative results from peer‐based, virtual interventions, and case management. In a Uganda‐based study evaluating HIVST distribution through trained community peer‐leaders, social network members reported that HIVST increased access to HTS, and the privacy and confidentiality of HIV test results [90]. A US‐based formative study evaluating the development of a smartphone application to support HIVST use among MSM and transgender women showed potential to address ongoing concerns about the use of HIVST, such as correct reading of results, and linkage to HIV care [89]. Lastly, in a Ukraine‐based strengths‐based case management study, interviewees saw linkage coordinators as accessible health specialists helping clients navigate the complicated healthcare system [88].

## 4 DISCUSSION

In this scoping review, we reviewed 66 studies, nine on linkage to HIV prevention and 57 on linkage to care. Despite the availability of a wide range of HIV prevention interventions, there is a dearth of literature on HIV prevention programmes and on the use of HIV messaging about the effectiveness of treatment for prevention [91]. Because no single HIV prevention intervention is sufficient to control HIV, prevention packages tailored to population needs are required.

Fifty‐seven linkage to care studies were identified, mainly from Africa and the Americas. Linkage to care studies focused primarily on streamlined interventions and case management and generally improved linkage to care and ART initiation. No studies were reported from the eastern Mediterranean region where only 41% of PLHIV knew their status, 27% of those aware were on ART and 24% of those on ART were virally suppressed as of 2021 [92].

Studies on streamlined interventions involved same‐day HIV testing, ART initiation, and POC CD4 and/or viral load testing and focused mainly on heterosexual African adults receiving community‐based testing, and on pregnant women and mothers receiving ART within integrated ANC settings. Streamlined interventions support WHO's recommendations on same‐day ART initiation among PLHIV, reducing the time to viral suppression [93]. However, some people may not want to initiate ART on the same day as their HIV diagnosis. Therefore, effective linkage to ART support services should be provided for PLHIV who re‐test positive in HTS [93]. Successful streamlined linkage to ART interventions could be leveraged to support same‐day PrEP initiation among higher‐risk individuals testing HIV negative. And because there was no difference in linkage to ART when comparing home‐based to mobile‐clinic‐based HTS, or before, during and after clinical consultation, there is an opportunity to diversify HTS settings to suit client preferences [42, 43].

Studies on HIV case management involved linkage coordinators developing personalized HIV care and risk reduction plans for clients mainly reported among key populations in the United States and Europe. Overall, HIV case management improved linkage to care and ART initiation highlighting the need for people‐centred care in HIV programmes [3]. However, while HTS for hospitalized patients is standard practice, intense inpatient case management may not be as effective in improving linkage to care compared to post‐discharge follow‐up, highlighting the importance of ongoing client support in the community [50]. People‐centred strategies were also favoured by clients receiving partner services, peer‐based, and multiple interventions that fast‐tracked HTS and ART initiation and provided ongoing counselling [13]. Such approaches provide ongoing support to higher‐risk HIV‐negative individuals seeking combination HIV prevention strategies based on their needs and perceived risk.

Studies evaluating virtual interventions showed mixed results. In a Thai study among MSMs, participants receiving online HIVST support were less likely to link to care compared to those receiving either a hybrid (offline HTS and online counselling) or in‐person HIV testing and counselling [78]. A US‐based study among prisoners using case management with video conferencing showed no impact on linkage to care [72]. In qualitative studies, participants found virtual interventions acceptable but preferred hybrid formats that incorporated direct human interaction [73]. Policymakers, therefore, may need to provide some elements of direct human interaction or innovative ways to continue follow‐up when designing virtual strategies for HIV negative.

While previous studies have confirmed that financial incentives may increase HIV testing uptake in the short‐term, in general, cash‐based incentives did not seem to consistently improve linkage to care or ART initiation [56, 57, 94]. While they were appreciated by participants, they elicited ethical discussions on who can be influenced by money and what can be bought if other alternatives to access healthcare were available, for example borrowing money for transport or walking to healthcare facilities [87]. WHO does not recommend financial incentives for clients in general because of cost, sustainability and ethical considerations.

QI studies, involving staff training to improve HTS delivery and data quality, did not seem to improve linkage to care or ART initiation [62, 63]. In these cluster‐RCT studies, there was a high risk of contamination if the staff shared QI strategies across different clusters. As no QI studies were reported on linkage to prevention, other research designs, for example pre‐post intervention studies, might be more helpful in reviewing the effectiveness of QI strategies.

Our scoping review has several limitations. First, we limited our search to articles written in English, potentially excluding articles in other languages and introducing bias in the geographical distribution of published research. Second, there was significant variation in the definitions of linkage to prevention and care and in delivery approaches applied across studies. Third, although our search strategy was extensive, this was not a systematic review; therefore, relevant articles could have been missed, and the methodological quality of the included studies such as the risk of bias was not assessed. Fourth, we excluded articles that focused solely on viral load suppression. However, the studies we reviewed on ART initiation may indicate interventions that support eventual viral suppression. Finally, there was a paucity of studies on linkage to screening for non‐HIV conditions, with only one reference on linkage to cervical cancer screening, indicating a gap for further research.

## 5 CONCLUSIONS

This scoping review provides an overview of linkage to prevention and care interventions. Despite a wide range of successful approaches to support linkage to care and ART, few studies provide global guidance on increasing linkage to prevention. As high‐burden countries reach the UNAIDS 95‐95‐95 targets, HIV incidence is declining but not sufficient to substantially reduce new acquisitions by 2030 [2]. Differentiated approaches for linkage to prevention, care and treatment are essential based on the epidemiological context, population group and individual risk. Although effective linkage to care interventions may offer insights on how to design combination HIV prevention packages, greater effort to generate evidence to optimize linkage to prevention is a global priority.

## COMPETING INTERESTS

The authors declare no competing interests.

## AUTHORS’ CONTRIBUTIONS

Conceptualization: C.J., M.J., N.F., R.B. and P.C., Data curation: B.W., P.C., C.J., N.S. and M.J., Formal analysis: B.W. and P.C., Methodology: P.C., B.W., N.S., M.J., N.F. and C.J., Project administration: B.W., Software: P.C. and B.W., Supervision: P.C., M.J. and C.J., Validation: B.W., P.C., M.J., C.J., N.S. and R.B., Visualization: B.W., Writing—original draft: B.W., P.C., C.J. and M.J., Writing—review and editing: B.W., P.C., M.J., N.S., R.B., N.F. and C.J.

## FUNDING

This work was funded by the Bill and Melinda Gates Foundation INV‐024432.

## DISCLAIMER

The funders had no role in study design, data collection and analysis, decision to publish or preparation of the manuscript. The views expressed in this article are those of the authors and do not represent the views of the WHO.

## PATIENT AND PUBLIC INVOLVEMENT

Patients and/or the public were not involved in the design, conduct, reporting or dissemination plans for this research.

## PROVENANCE AND PEER REVIEW

Not commissioned, externally peer‐reviewed.

## Supporting information


**Appendix 1**: Search components

**Supporting Information**: Preferred Reporting Items for Systematic reviews and Meta‐Analyses extension for Scoping Reviews (PRISMA‐ScR) Checklist

## Data Availability

Data sharing is not applicable to this article because it is a review, and no new data were generated during the current study. The protocol is available on request from the corresponding author.
