# Supplementary material for: Understanding effective post‐test linkage strategies for HIV prevention and care: a scoping review
Source: J Int AIDS Soc. 2024 Apr 11;27(4):e26229. doi: 10.1002/jia2.26229 (PMC11009370; doi:10.1002/jia2.26229)
Supplement: Supplementary file 1 — Appendix 1: Search components [file JIA2-27-e26229-s001.docx]

**Appendix 1: Search components**

Component 1a: HIV testing

("HIV Infections/diagnosis"[Mesh] OR "AIDS Serodiagnosis"[Mesh]) OR (("HIV Infections"[Mesh] OR “HIV”[mesh] OR HIV[tiab] OR HIV1[tiab] OR HIV2[tiab] OR “human immunodeficiency virus” [tiab] OR “human immunedeficiency virus” [tiab] OR “human immuno deficiency virus” [tiab] OR “human immune deficiency virus” [tiab] OR ((human immun*[tiab]) AND (deficiency virus[tiab])) OR "Acquired Immunodeficiency Syndrome"[Mesh] OR ((acquired immun*[tiab]) AND (deficiency syndrome[tiab])) OR “acquired immunodeficiency syndrome”[tiab] OR “acquired immunedeficiency syndrome”[tiab] OR “acquired immuno-deficiency syndrome”[tiab] OR “acquired immune-deficiency syndrome”[tiab]) AND ("Diagnostic Tests, Routine"[Mesh] OR "Mass Screening"[Mesh] OR diagnos*[tw] OR serodiagnos*[tw] OR test[tiab] OR tests[tiab] OR testing[tiab] OR screening[tiab]))

Component 1b: Linkage/Care/Prevention

(Link [tw] OR linkage[tw] OR link* to care [tw] OR link* to prevent* [tw] OR enrol* to care [tw] OR consult* to care[tw] OR access* to care[tw] OR access* care[tw] OR engag* to care[tw] OR connect* to care[tw] OR enter* to care[tw] OR entry to care[tw] OR initiat* to care[tw] OR initiat* care[tw] OR integrat* to care[tw] OR integrat* care[tw] OR attend* care[tw] OR enrol* to prevent* [tw] OR prevent* consult*[tw] OR access* to prevent*[tw] OR access* prevent*[tw] OR engag* to prevent*[tw] OR connect* to prevent*[tw] OR enter* to prevent*[tw] OR entry to prevent*[tw] OR initiat* prevent*[tw] OR prevent* initiat* [tw] integrat* prevent*[tw] OR “hospital discharge”[tw] OR “prison release”[tw] OR “jail release”[tw] OR “prison release” [tw])

Component 2a: Peer-based

("Patient Navigation"[Mesh] OR “Social Support”[mesh] OR mentor* [tiab] OR norm[tiab] OR norms [tiab] OR norming[tiab] OR navigation[tiab] OR “self help”[tiab] OR peer[tiab] OR peers[tiab] OR friend[tiab] OR friends[tiab] OR “social network”[tiab] OR “social networks”[tiab] OR “support group”[tiab] OR “support groups”[tiab])

Component 2b: Incentives

("Reward"[Mesh] OR “Remuneration”[Mesh] OR “Health services needs and demand/economics”[Mesh] OR “Health promotion/economics”[Mesh] OR “Economics, medical”[Mesh] OR “Education/economics”[Mesh] OR “Financial Support”[Mesh] OR “health services accessibility/economics”[Mesh] OR "Financing, Personal"[Mesh] OR Reimburs*[tiab] OR demand [tiab] OR demands[tiab] OR incentiv* [tiab] OR payment* [tiab] OR voucher* [tiab] OR payment [tiab] OR lottery[tiab] OR lotteries[tiab] OR “cash”[tiab] OR compensat*[tiab] OR reward*[tiab] OR prize*[tiab] OR remunerat*[tiab] OR “cash transfer”[tiab] OR “cash transfers”[tiab] OR monetary[tiab] OR money[tiab] OR “financial compensation”[tiab] OR “economic compensation”[tiab] OR “performance based”[tiab])

Component 2c: SMS and digital individual media

("Cell Phone"[Mesh] OR "Computers, Handheld"[Mesh] OR “Internet”[mesh] OR “Wireless Technology”[mesh] OR “Telephone”[mesh] OR "Social Media"[Mesh] OR "Electronic Mail"[Mesh] OR "Text Messaging"[Mesh] OR “social media”[tiab] OR “social networking”[tiab] OR crowdsourc*[tiab] OR texting[tiab] OR “text message”[tiab] OR “text messages”[tiab] OR “text messaging”[tiab] OR “short messaging service”[tiab] OR “short message service”[tiab] OR “sms message”[tiab] OR “sms messages”[tiab] OR “sms messaging”[tiab] OR “instant message”[tiab] OR “instant messages”[tiab] OR “instant messaging”[tiab] OR “phone”[tiab] OR “phones”[tiab] OR “mobile technology”[tiab] OR “mobile device”[tiab] OR “mobile devices”[tiab] OR “cellular technology”[tiab] OR smartphone*[tiab] OR telephone*[tiab] OR internet[tiab] OR wireless[tiab] OR whatsapp[tiab] OR twitter[tiab] OR video[tiab] OR videos[tiab] OR android[tiab] OR “operating system”[tiab] OR “mobile health”[tiab] OR mhealth[tiab] OR “m health”[tiab] OR ehealth[tiab] OR “e health”[tiab] OR computer[tiab] OR computers[tiab] OR website*[tiab] OR “web site”[tiab] OR “web sites”[tiab] OR internet[tiab] OR online[tiab] OR “online chat”[tiab] OR “chat room”[tiab] OR “chat rooms”[tiab] OR email[tiab] OR “e mail”[tiab] OR “electronic mail”[tiab] OR facebook[tiab])

Component 2d: Streamlined interventions

("streamline"[All Fields] OR "streamlined"[All Fields] OR "streamlining"[All Fields]) AND ("intervention s"[All Fields] OR "interventions"[All Fields] OR "methods"[MeSH Terms] OR "methods"[All Fields] OR "intervention"[All Fields] OR "interventional"[All Fields]) AND ("hiv testing"[MeSH Terms] OR ("hiv"[All Fields] AND "testing"[All Fields]) OR "hiv testing"[All Fields]) AND "linkage"[All Fields]) OR "linkage"[All Fields] OR "linkages"[All Fields])

Component 2e: Case management

("case management"[MeSH Terms] OR ("case"[All Fields] AND "management"[All Fields]) OR "case management"[All Fields]) AND ("hiv testing"[MeSH Terms] OR ("hiv"[All Fields] AND "testing"[All Fields]) OR "hiv testing"[All Fields]) AND "linkage"[All Fields]) OR "linkage"[All Fields] OR "linkages"[All Fields])

Component 2f: Tracing

("trace"[All Fields] OR "traced"[All Fields] OR "tracing"[All Fields] AND ("hiv testing"[MeSH Terms] OR ("hiv"[All Fields] AND "testing"[All Fields]) OR "hiv testing"[All Fields]) AND "linkage"[All Fields]) OR "linkage"[All Fields] OR "linkages"[All Fields])

**Supporting information**

Appendix S1. PRISMA scoping review (ScR) checklist.
